# Adaptation and validation of the Berlin questionnaire of competence in evidence-based dentistry for dental students: a pilot study

**DOI:** 10.1186/s12909-020-02053-0

**Published:** 2020-05-04

**Authors:** Laura Imorde, Andreas Möltner, Maren Runschke, Tobias Weberschock, Stefan Rüttermann, Susanne Gerhardt-Szép

**Affiliations:** 1 Department of Operative Dentistry, Dental School (Carolinum), Goethe-University Frankfurt, Theodor-Stern-Kai 7/29, D-60596 Frankfurt am Main, Germany; 2 Center of Excellence for Assessment in Medicine, University of Heidelberg, Heidelberg, Germany; 3 Institute of General Practice, Goethe University Frankfurt, Frankfurt am Main, Germany; 4 Department for Dermatology, University Hospital Goethe University Frankfurt, Frankfurt am Main, Germany

**Keywords:** Evidence-based medicine, Evidence-based dentistry, Evaluation, Dental practice, Reliability, Questionnaire, Validation

## Abstract

**Background:**

The purpose of this pilot study was to create a valid and reliable set of assessment questions for examining Evidence-based Dentistry (EbD) knowledge. For this reason, we adapted and validated for dental students the Berlin Questionnaire (BQ), which assesses Evidence-based Medicine (EbM) abilities.

**Methods:**

The Berlin Questionnaire was validated with medical residents. We adapted it for use in a dentistry setting. An expert panel reviewed the adapted BQ for content validity. A cross-sectional cohort representing four training levels (EbD-novice dental students, EbD-trained dental students, dentists, and EbM−/EbD-expert faculty) completed the questionnaire. A total of 140 participants comprised the validation set. Internal reliability, item difficulty and item discrimination were assessed. Construct validity was assessed by comparing the mean total scores of students to faculty and comparing proportions of students and faculty who passed each item.

**Results:**

Among the 133 participants (52 EbD-novice dental students, 53 EbD-trained dental students, 12 dentists, and 16 EbM-/ EbD-expert faculty), a statistically significant (*p* < 0.001) difference was evident in the total score corresponding to the training level. The total score reliability and psychometric properties of items modified for discipline-specific content were acceptable. Cronbach’s alpha was 0.648.

**Conclusion:**

The adapted Berlin Questionnaire is a reliable and valid instrument to assess competence in Evidence-based Dentistry in dental students. Future research will focus on refining the instrument further.

## Background

Evidence-based Medicine (EbM) represents the practical search for and the application of the best available evidence to act properly in clinical situations [1–4]. Practising EbM combines individual internal clinical expertise with external evidence from systematic research and applies it to make decisions for individual patients [1]. The term EbM was officially introduced in 1992 to guarantee good and scientifically valid treatment for patients [5, 6]. Since then, many different practices to teach EbM, as well as assessment procedures for measuring EbM skills [5, 6], have been introduced. To better evaluate these practices, comprehensive, valid and practical instruments to assess and measure EbM knowledge [6–9] are needed.

Although many instruments exist for evaluating EbM curriculum effectiveness [5–7], only two − the Fresno Test (FT) [8] and the Berlin Questionnaire (BQ) [7, 9] − are well represented. These two tests have been frequently tested and show good validity and reliability and they cover a broad range of EbM knowledge and skills [2, 5–7]. Both tests are defined as Level 1 instruments, which are characterised by the capability to discriminate between different training levels or performances [7]. Levels 2 and 3 have lower psychometric properties [7].

The FT is distinguished by performing realistic Evidence-based Practice (EbP) tasks. In comparison to the multiple-choice format of the BQ, the FT requires more time and expertise to grade [7]. The BQ is not as extensive as the FT for assessing applied EbM knowledge, which makes grading easier to perform [7]. Both the FT [8] and the BQ [9] represent the only instruments that evaluate four of five EbM steps (asking, accessing, appraising and applying) [7]. The BQ contains multiple-choice questions on typical clinical scenarios. The questions cover a broad range of epidemiological knowledge, statistics and concepts that refer to Evidence-based Medicine in the clinical context; for example, diagnostic tests and systematic reviews. It consists of two parts (parts A and B) so that changes in subjects’ knowledge as a result of an intervention can be determined (for example, before and after a course) [9]. The BQ has good psychometric values, such as the excellent statistically determined values of internal consistency, difficulty and ability to discriminate. Thus, the BQ represents a high-quality detection tool for EbM knowledge [5, 7, 9].

The majority of published instruments, including the BQ, address the medical arena without dental relevance. Dental evaluation tools are valuable because Evidence-based Dentistry is becoming increasingly important. A large amount of information is available to dentists, which makes treating patients more challenging [4, 10]. The ability to identify, appraise and apply biomedical research is an essential skill for clinical decision making [11–13] − not only for medical doctors but also for dentists in their role as dental experts [12, 14, 15]. Therefore, teaching and measuring Evidence-based Dentistry is necessary to enable dental students to become evidence-based working professionals with a high degree of expertise [14–17]. Currently, EbD knowledge and practice among students seem to be deficient [18]. The issue of EbD is also enshrined in the current version of licensing of dentists [19]*,* as well in the National Competency-Based Learning Objective Catalogue Dentistry [20]. To be able to assess competency in EbD, the dental profession needs an instrument that is valid and reliable.

For this reason, similar to other studies [2, 6, 17], we performed an adaptation and validation process of an already examined questionnaire to create a new form for the target group of dental students/ dentists. We chose the BQ (here called Berlin Questionnaire Medical = BQM) as a starting point for our instrument because rather than open-ended responses, it requires only the identification of the correct answer from a list. Further reasons are explained under the point discussion. The questionnaire had been developed and validated to assess medical residents’ competence [9]. However, the BQM has not been validated for assessing dental students’ competence.

The main goal of this pilot study was to create a modified Berlin Questionnaire that is just as meaningful as the original BQ for use with dental residents. After testing, the expected test scores should be analysed to ensure the validity of the questionnaire. The conceptual framework of this pilot study was based on the classic quality criteria of assessment tools: reliability and validity. In the analysis of the results, we measured reliability − which indicates how accurate the results are − by using Cronbach’s alpha. We used validity to indicate whether that which is being measured is what ought to be measured. The different forms of validity are shown below. First, content validity ensures that the semantic correspondence between the present measuring instrument and the construct are checked for plausibility. Content validity is either obvious (face validity) or is assessed as valid by experts (expert validity), as we have planned in this pilot study. Second, construct validity measures the conformity between the constructs and their measurements and was interpreted in the evaluation of the results with the known groups method. Third, criterion validity is the measurement of an appropriate comparison criterion that represents the base. This form of validity could not be carried out in the present study because, for example, the original questionnaire would have had to be used as a comparative value. This is noted in the outlook at the end. Correspondingly, the aim of good assessment tools is high validity and high reliability. An examination tool that is not reliable is also not valid [21].

## Methods

### Development of the adapted Berlin questionnaire

**Fig. 1:**
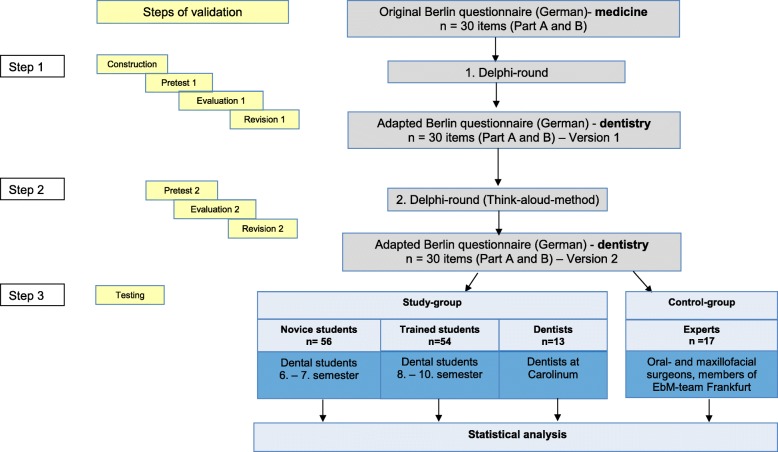
Steps of validation

The original Berlin Questionnaire contains 15 items [9, 19]. This instrument was adapted for use with dental students. It was designed, adapted and validated in three steps: 1) discipline-specific modification, 2) establishment of content validity and 3) testing as already described by Tilson [6]. The steps of validation are explained below and demonstrated in Fig. 1.

#### Step 1: Construct a discipline-specific questionnaire

**Fig. 2:**
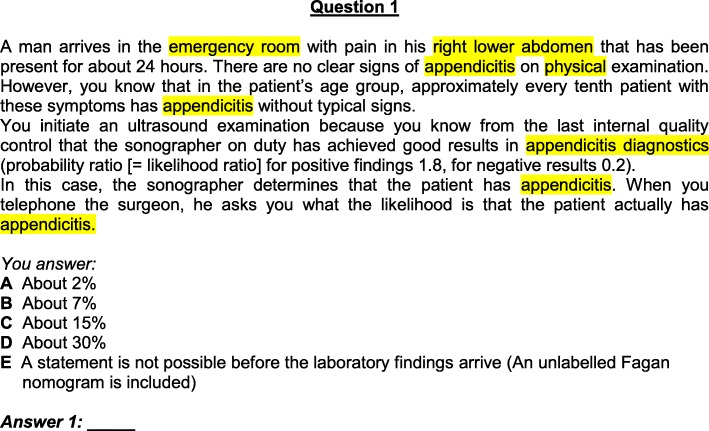
Original question A1

**Fig. 3:**
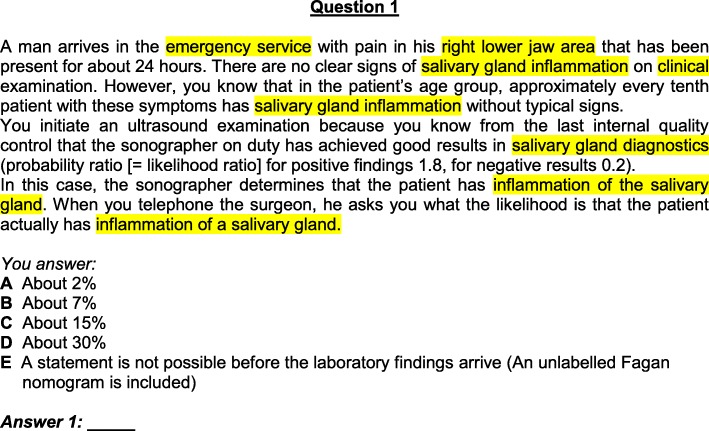
Adapted question A1

In the first step, the first round of Delphi, all questions were reworked from the medical context into a dental setting as illustrated in Figs. 2 and 3.

The adapted instrument was given the title “Berlin Questionnaire of Dental Competence in Evidence-based Dentistry.” In the following article, the questionnaire is referred to as the Berlin Questionnaire Dental (BQD). Table 1 compares the original Berlin items with the adapted items.

**Table 1:** Comparison of items in part A and B

	Original items of the Berlin-Test	Adapted items
A1	emergency room	emergency service
	lower abdomen	lower jaw
	appendicitis	inflammation of the salivary duct
A2	appendicitis	inflammation of the salivary duct
A3	appendicitis	inflammation of the salivary duct
A4	lipid reducers	medication
	heart attack	salivary gland tumour (mucoepidermoid tumour)
A5	no change	
A6	headache	toothache
A7	headache	toothache
A8	slimming pills	antibacterial mouth rinsing solution
	cardiovascular mortality	bacterial endocarditis
	overweight patients	risk patients
	lactose pill (placebo)	mouth rinse (placebo)
A9	slimming pills	antibacterial mouth rinsing solution
	cardiovascular death	endocarditis death
A10	internist	medical specialist for oral and maxillofacial surgery
	carotid artery	facial artery
	stenosis of the carotid artery	stenosis of the facial artery
A11	lipid reducers after myocardial infarction	medication after cancer of mouth base
	adjusted hypertension (RR: 155/98 mmHg) and obesity	normal oral hygiene and tobacco use
	deathly infarction	deathly outcome
	heart attack	cancer of mouth base/ death
A12	gastro-enterological policlinic	oral surgery polyclinic
	colon cancer	squamous cell carcinoma
A13	breast	vestibulum
	gynaecologist	oral- and maxillofacial surgeon
	breast cancer	cancer
	gynaecological ambulance	oral- and maxillofacial ambulance
	mammograms	radiographs
A14	leg vein thrombosis	vein thrombosis
	You would like to know …	You would like to know for reasons of personal interest …
A15	no change	

In the first round of Delphi, the first version of the BQD was designed. The implementation involved two experts. The two experts were experienced with Evidence-based Dentistry having achieved pre-doctoral as well as master’s degree education. They wrote e-mails about their modifications and discussed them. This started the implementation of the discussed points. The content validity was ensured by the dental expertise of the two authors.

#### Step 2: validate the modified questionnaire

In the second Delphi round, the procedure followed the same pattern. The first version of the questionnaire was pre-tested, evaluated and revised. The evaluation was conducted using the think-aloud- method. In this method, people are asked to verbalise their thinking during the thought process [22]. This technique should reveal differences in specific thinking as well as thoughts related to expertise and lead to a better design of the items [23]. The second Delphi round was then conducted by nine experts (panellists 1 to 3: pre-doctoral education with a low degree of EbD expertise; panellists 4 and 5: pre-doctoral education with a middle degree of EbD expertise; panellist 6: pre-doctoral education with high EbM/EbD expertise; panellists 7 and 8: Master’s degree education; panellist 9: doctoral education).

Panellist feedback addressed comprehensibility and item clarity. The proposed amendments included changes in paragraphs and the use of alternative vocabulary to achieve better understanding. The panel also suggested some structural modifications and alterations to items to guarantee a clear setting. This step of development guaranteed the objectivity of interpretation.

The new dental version of the test contains 15 items in part A and 15 in part B. The complete test and scoring rubric are shown in the Additional file 1.

#### Step 3: Test the modified questionnaire

After the second round of Delphi, the modified questionnaire was distributed to the study group (*n* = 240) and the control group (*n* = 17) for processing. The study group included novice students (*n* = 87) in the sixth to seventh semesters, and a group of trained students (*n* = 140) in the eighth to tenth semesters. The group of dentists consisted of 13 dentists. The expert group was composed of student trainers from the EbM Team Frankfurt am Main and maxillofacial surgeons (*n* = 17). The EbM Team Frankfurt is a cooperation of the Goethe-University that is committed to improving the application of EbM. Since 2003, they have run the compulsory Evidence-based Medicine seminars for medical students in a peer-teaching approach.

This pilot study was approved by the University of Frankfurt Institutional Board, and all the participants gave informed consent to participate.

### *E*xamination sample

All the students in the study group were enrolled at the Goethe-University of Frankfurt. Inclusion criteria included belonging to one of the following groups: Sixth- to tenth-semester dentistry students at the Goethe-University of Frankfurt, dentists in the dental school, maxillofacial surgeons of the Goethe-University of Frankfurt or members of the EbM Team Frankfurt. The distribution of the study population was represented as follows: 24 participants were in the sixth semester, 32 participants in the seventh semester, 17 participants in the eighth semester, 26 participants in the ninth semester, and 11 participants in the tenth semester. The group of dentists consisted of 13 participants. The group of maxillofacial surgeons were 5 participants, and the members of the EbM- Team comprised 12 participants. In each group, the number of female attendees was higher than the number of male participants. Except for the maxillofacial surgeon group.

### Setting

The participants received no verbal instruction. The text on the first page of the test contained the necessary instructions. The maximum processing time was 45 min. All questionnaires were processed on the premises of the Goethe-University of Frankfurt. The entire question pool was answered in one session. No intervention was made between part A and part B.

### Data analysis

Data were analysed using R version 3.5.1 and SAS 9. Before evaluating the test results, all the participants who did not answer more than seven out of 30 questions were removed from the rating [24].

### Total score reliability

To assess the reliability and reproducibility of the test, Cronbach’s alpha was determined.

### Individual item analysis

Statistical analyses of the test tasks included item difficulty and item discrimination power for each question. Item difficulty was estimated using the item mean. We categorised the measurements as follows: Very easy tasks were defined as having values of *P* ≥ 0.85, medium-heavy tasks with values of 0.85–0.40 and heavy tasks with a value of *P* ≤ 0.40. Tasks with good discriminatory power showed values of r’ ≥ 0.20 [21].

### Differences among groups

The results of the study- and control- groups were analysed and checked for a significant difference to ensure the construct validity. This was done by variance analysis with factor groups (novice students, trained students, dentists, experts) and Tukey-Kramer post hoc comparisons.

### Exploratory factor analysis

An exploratory factor analysis (maximum-likelihood factor analysis) was carried out to analyse the structure of the BQD. Likelihood-ratio tests were used to determine the number of factors. This factor analysis is based on the Pearson correlation coefficients. A second factor analysis based on tetrachoric correlations was carried out for control purposes.

## Results

### Dropout rate

**Fig. 4:**
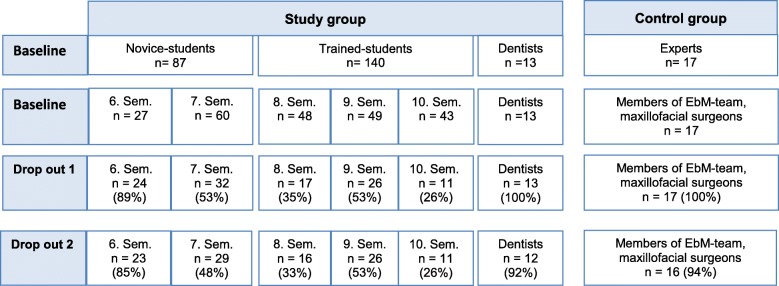
Dropout rate

Figure 4 demonstrates a two-stage dropout procedure. A total of 257 persons with different levels of EbD- knowledge were invited to participate. However, not all of the requested students wanted to participate. The BQD was edited by 140 participants (56 novice dental students, 54 trained dental students, 13 dentists and 17 experts). Seven participants were excluded from the evaluation to reduce the risk of distorting the outcome. Those seven participants did not answer eight or more of the 30 questions. The mean dropout rate was 51.75%. The mean response rate for the total study population was 48.25%.

### Total score reliability

The reliability of the questionnaire was described by Cronbach’s alpha. The value of Cronbach’s alpha for the complete test including part A (*n* = 15 items) and part B (*n* = 15 items) was 0.648 (95% confidence interval: 0.556–0.729). For the single parts, the value of Cronbach’ alpha for part A was 0.382 (95% confidence interval: 0.217–0.526), and part B was 0.533 (95% confidence interval: 0.409–0.642). Parts A and B correlated significantly (*p* < 0.001) to 0.518 (95% confidence interval: 0.382–0.633).

The numeric values of Cronbach’s alpha for the different groups with regard to parts A and B were 0.5065 (95% confidence interval: 0.237–0.095) for the novice dental students, 0.5140 (95% confidence interval: 0.252–0.070) for the trained dental students and 0.8245 (95% confidence interval: 0.490–0.037) for the dentists. The control group reached an alpha value of 0.8888 (95% confidence interval: 0.732–0.500).

### Individual item analysis

Table 2 describes the results for each question of the test. The average, standard deviation, item difficulty and discrimination power were examined for each question of the test. The analysis showed that 28 of 30 questions had a *p*-value < 0.40. In addition, 15 of the 30 questions showed a discriminatory power of <0.20.

**Table 2:** Individual item analysis

Question	Points	Average	Standard deviation	Difficulty	Discriminatory power
Test	0–30	8.421	3.962	0.281	–
A1	0–1	0.353	0.480	0.353	0.126
A2	0–1	0.143	0.351	0.143	0.042
A3	0–1	0.256	0.438	0.256	−0.120
A4	0–1	0.398	0.491	0.398	0.403
A5	0–1	0.263	0.442	0.263	0.216
A6	0–1	0.353	0.480	0.353	0.052
A7	0–1	0.263	0.442	0.263	0.337
A8	0–1	0.391	0.490	0.391	0.033
A9	0–1	0.195	0.398	0.195	−0.023
A10	0–1	0.338	0.475	0.338	0.218
A11	0–1	0.203	0.404	0.203	0.039
A12	0–1	0.346	0.447	0.346	0.504
A13	0–1	0.256	0.438	0.256	0.059
A14	0–1	0.278	0.450	0.278	0.160
A15	0–1	0.361	0.482	0.361	0.421
B1	0–1	0.316	0.467	0.316	0.115
B2	0–1	0.353	0.480	0.353	0.338
B3	0–1	0.256	0.438	0.256	0.573
B4	0–1	0.398	0.491	0.398	0.167
B5	0–1	0.278	0.450	0.278	0.205
B6	0–1	0.241	0.429	0.241	0.259
B7	0–1	0.098	0.298	0.098	0.194
B8	0–1	0.158	0.366	0.158	−0.008
B9	0–1	0.444	0.499	0.444	0.421
B10	0–1	0.406	0.493	0.406	0.395
B11	0–1	0.143	0.351	0.143	0.098
B12	0–1	0.256	0.438	0.256	0.278
B13	0–1	0.241	0.429	0.241	−0.039
B14	0–1	0.271	0.446	0.271	0.170
B15	0–1	0.165	0.373	0.165	0.049

### Known groups’ validity

**Fig. 5:**
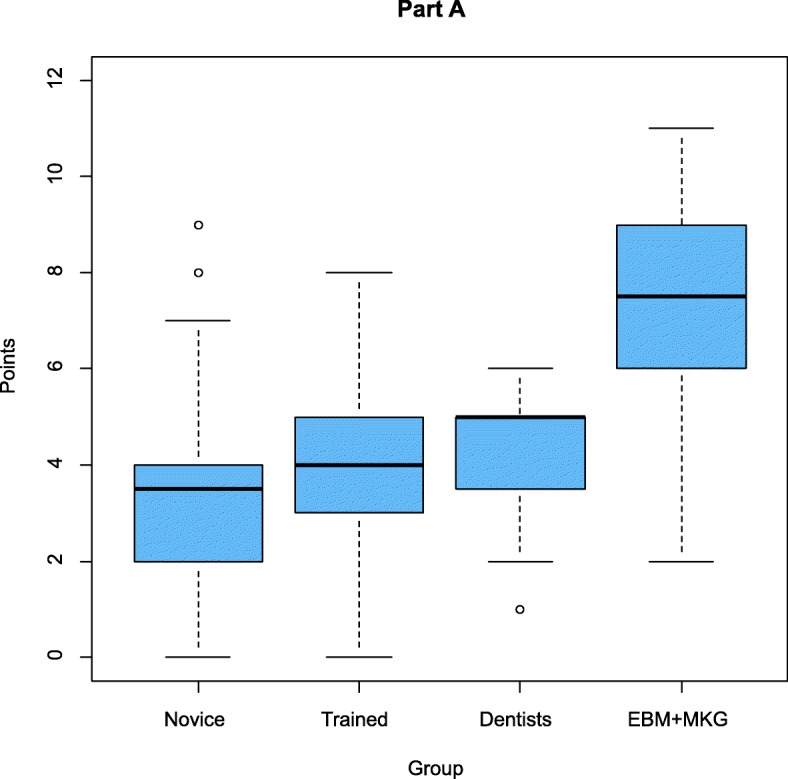
Scores for the Berlin Questionnaire Dental by groups Part A

**Fig. 6:**
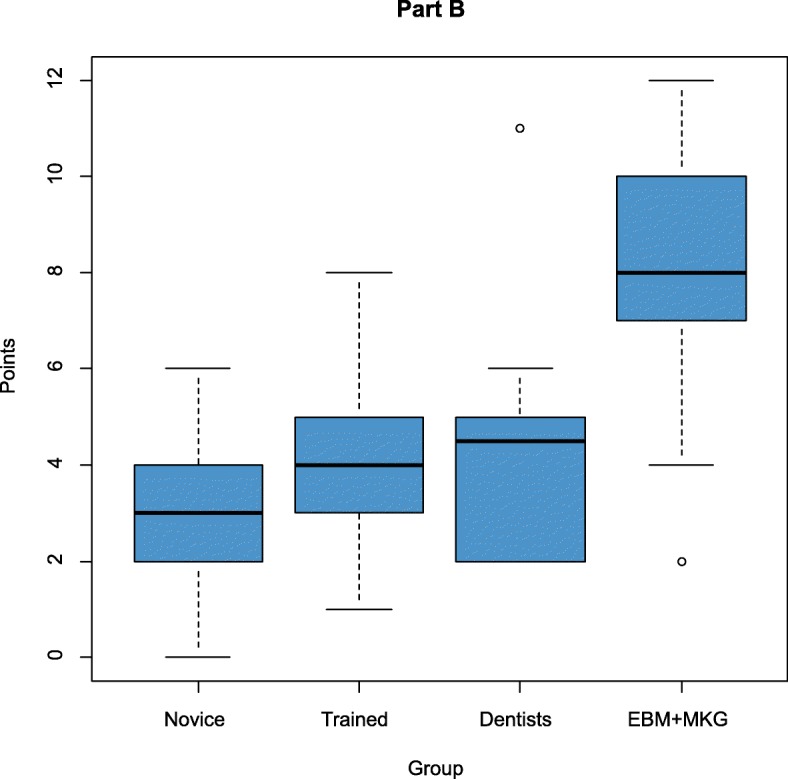
Scores for the Berlin Questionnaire Dental by groups Part B

**Fig. 7:**
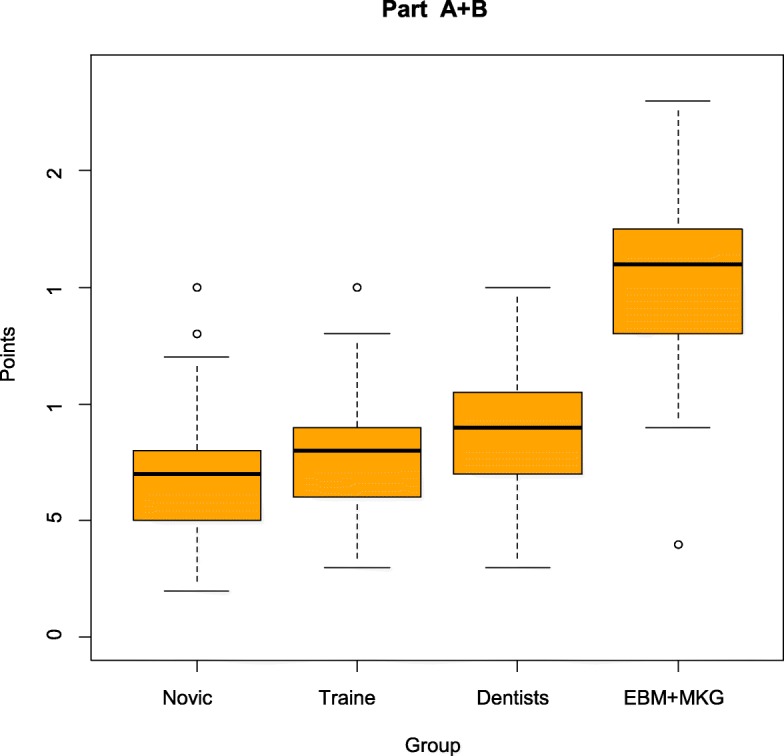
Scores for the Berlin Questionnaire Dental by groups Part A + B

The results of the four groups (novice students (group 1), trained students (group 2), dentists (group 3) and experts (group 4)) were analysed and compared (Fig. 5, 6 and 7, Tables 3 and 4). The maximum attainable point value was 30 points. The average of the points reached in group 1 was 6.73. Group 2 achieved a value of 7.98 and group 3 a value of 8.58 The expert group demonstrated an average value of 15.25. The control-group was significantly different compared to the study-group (*p* < 0.001).

**Table 3:** ANOVA table

	Df	Sum Sq	Mean Sq	F value	Pr (>F)	
**Group**	3	905.29	301.764	33.353	*p* < 0.001	
**Residuals**	129	1167.13	9.048			
**Group 1**	**Group 2**	**Mean 1**	**Std.-dev. 1**	**Mean 2**	**Std.-dev. 2**	**Cohens d**
Dentists	EBM + MKG	8.583	3.288	15.250	4.612	1.688
Dentists	Novice	8.583	3.288	6.731	2.568	0.633
Dentists	Trained	8.583	3.288	7.981	2.749	0.200
EBM + MKG	Novice	15.250	4.612	6.731	2.568	2.373
EBM + MKG	Trained	15.250	4.612	7.981	2.749	1.975
Novice	Trained	6.731	2.568	7.981	2.749	0.470

**Table 4:** Pairwise comparison of groups (Tukey-Kramer tests)

Contrast	Estimate	SE	df	t.ratio	*P*.value
Dentists - EBM + MKG	−6.667	1.149	129	−5.804	<.0001
Dentists - Novice	1.853	0.963	129	1.923	0.2235
Dentists - Trained	0.602	0.962	129	0.626	0.9235
EBM + MKG - Novice	8.519	0.860	129	9.907	<.0001
EBM + MKG - Trained	7.269	0.858	129	8.472	<.0001
Novice - Trained	−1.250	0.587	129	−2.130	0.1492

The standard deviation of the first two groups was 2.6. The dentists showed a standard deviation of 3.3 and the experts 4.6.

### Exploratory factor analysis

**Fig. 8:**
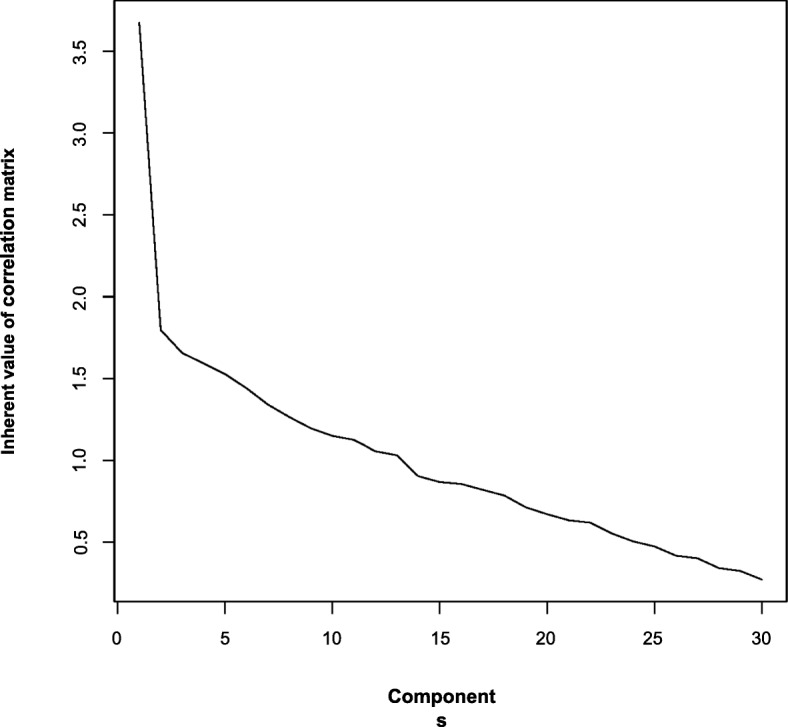
Scree plot

The exploratory factor analysis showed no subclasses within the questionnaire. It presented a single factor test (Fig. 8). The Bartlett test of the hypothesis that at least one factor was included in the questionnaire, was significant at *p* < 0.001 (chi^2^ = 622.60, df = 435). The likelihood ratio test for whether more than one factor was included was not significant with *p* = 0.439 (chi^2^ = 437.91, df = 434). The factor analysis based on tetrachoric correlations gave the same result.

## Discussion

This pilot study proves that the adapted BQ is a valid and reliable instrument to use with dental students for measuring EbD knowledge and abilities. At the current time, this is, to the best of our knowledge, the first adapted and valid Berlin Questionnaire that assesses EbD knowledge and skills. In the current literature, numerous instruments can be found that assess EbM knowledge [8, 9, 25–27]. Strongly represented and well-studied are the following three tools: the Fresno Test [8], the Berlin Questionnaire [7, 9] and the ACE (Assessing Competency in EbM) tool [28]. Many of these tools have been adapted, modified and validated for other areas of application, such as pharmacy [2], physical therapy [6] and surgery [27]. Validation processes for other languages ​​have also been performed, such as by Spek [17] who translated the FT into Dutch, and by others [17, 29, 30]. Based on our search on the PubMed database in 2019, we observed that the field of questionnaires that specialised in measuring EbD knowledge is small.

### Type of questionnaire

Similar to many others [5, 26, 27, 29], we used the BQ as a starting point because it has a reliable, valid and objective construction [5, 7, 9, 26, 31]. The BQ has already been modified for specialized fields of medicine such as surgery by Ubbink [27], because its good critical appraisal of study design and interpretation of study results. We decided to use the multiple-choice format of the BQ instead of the free text answers of the FT or the binary answer format of the ACE tool [29]. The ACE tool, which uses a dichotomous question type, yields high inaccuracy due to 50% success from the participant guessing the right answer [29]. In the BQ, the participant has to choose one of five answer options, which makes the effect of random guessing smaller [29]. In comparison to the BQ and ACE tools, the open answer questions of the FT query a larger EbM range, but are more difficult and more time-consuming to grade [7]. Qureshi [32] adapted the Fresno Test for dental students, in which problems with a subjective evaluation of free answers were noticed. The questionnaire that Qureshi used is not available for free viewing. The questionnaire chosen by us provides a combination of manageable effort and accuracy of assessment, as already mentioned by different research groups [7, 29].

### Process of validation

The process of validation, as described in Methods, is comparable to Tilson’s study, including discipline-specific modification, the establishment of content validity and testing [6, 33]. Similar to Tilson, Spek and many others, we considered that a clinical scenario that fits into the target group of the evaluation instrument is the most authentic and effective [17]. For this purpose, we adapted the human medical context for the dental field. Since even minor changes to the questionnaire can affect its reliability and validity, we limited ourselves to the discipline-specific adaptation of the existing questions [17]. In contrast, Tilson created additional two completely new items. Fifty per cent of these are not further recommended, because of their questionable psychometric values [6].

### Validity

To ensure the construct validity, we used the known groups method, similar to Spek [17]. The results for the study-group versus the control-group (novice students, trained students and dentists versus experts) were significantly different and corresponded to the level of training (*p* < 0.001). This was also noted by Tilson on modifications of the Fresno Test [6]. The lack of significant differences within the study-group was not surprising due to the minimal proportion of EbD teaching in dentistry studies at Frankfurt am Main. Following Kortekaas and other studies describing the measurement of construct validity [25, 27, 28], the significance between the study-group and control-group demonstrated good construct validity of the BQD.

The scores show an increasing trend with higher knowledge levels of the participation group. Fritsche and West also recorded an increasing median score of the BQ depending on the education level [9, 26, 27]. The presented median scores in the study of West are similar to our results [26]. It is essential to take into account that the different results between the groups may have been affected by clinical experience [6]. The EbM/EbD expert cohort consisting of maxillofacial surgeons and members of the EbM Team Frankfurt showed (*p* < 0.001) better results than the study-group. The dentists of the study-group had already worked with patients. This suggests that EbD knowledge and skills are affected not only by clinical experience, according to Tilson [6], but also by medical education, including curricular EbM courses.

### Reliability

The values of Cronbach’s alpha of the BQD are acceptable and comparable to the study of Lai and Buljan, in which an adapted BQ was also tested [5, 29, 34, 35]. The BQ used in Lai’s study achieved a Cronbach’s alpha value of 0.7 [5]. In Buljan’s study, the questionnaire was translated into Croatian and reached an alpha value of 0.63 [29]. Fritsche used the original BQ, which reached Cronbach’s alpha values ​​of 0.75 for part A and 0.82 for part B [9]. This deviation of the alpha value is due to Spek’s statement that even small changes to the questionnaire can change its reliability [17]. If the questionnaire should also be used in state examinations, more items would be needed to get a higher value of Cronbach’s alpha [36, 37]. To determine the number of items that would be necessary to obtain a reliability of at least 0.8, the Spearman-Brown-formula can be used. For α = 0.648 a total number of 66 items is needed for a reliability of 0.8.

### Individual items: discipline-specific modification

The questions of the BQD were mostly difficult, just as in Buljan’s study [29]. The study by Tilson, who assessed the difficulty of the tasks of the Fresno Test by their pass rate, also had predominantly difficult questions [6]. In Ramos’ study, which also examined the Fresno Test, there were likewise moderate to difficult items, but no simple items available. This allowed the questionnaire to be compatible with different training levels [8]. Item difficulty is not only a characteristic of the question but also of the sample that is being tested [38]. The item difficulty can be used to interpret the overall result of the study population. For example, consistently poor answers to questions provide valuable information about knowledge gaps [38]. The item difficulty analysis would thus substantiate the suspicion that the tested population had received little education and training in EbD, which reflected the educational situation of our dental cohort.

In addition to the difficulty, the discrimination capacity of a task is relevant. According to Rush [39], 19 of 30 questions of the BQD have acceptable discriminatiory power. The discrimination of a task indicates the extent to which the success of the candidate is related to the overall success of the exam [39]. Items with a discrimination value of less than 0.10 or negative values should be checked to see whether the item is flawed [39]. In the BQD, nine questions were to be checked for discrimination deficiencies. In contrast, Ramos’ study on the Fresno Test showed no negative or low discrimination values [8]. Referring to the fact that the analysis of item difficulty already showed that the items were too difficult for the study-group, we draw a parallel with item discrimination. Because the tasks were too difficult, the study-group had to guess, which led to worse discrimination values.

### Exploratory factor analysis

The studies by Lai and Buljan show that the original Berlin questionnaire (part A) covers five domains [5, 29]. These mental constructs include study design, internal validity, the magnitude of effect/clinical importance, application and diagnostic accuracy. Due to the explorative factor analysis that we performed, we could not detect any subgroups in our questionnaire. We cannot confirm this factor structure with our data shown by Lai and Buljan.

### Limitations

The study was a pilot study using adapted instruments. Our goal was to make the BQ for human physicians applicable for EbD while maintaining its proven psychometric values already demonstrated by Fritsche [9].

### Participation

The total number of participants in this pilot study was 257 persons. After two dropout phases, the study population was comprised of 133 participants. Other similar study designs worked with comparable participant numbers. Coppenrath had a total of 120 participants [2], Buljan worked with a group of 91 participants [29], Tilson’s study worked with a questionnaire answered by 108 persons [6] and Lai used a participation group of 72 persons [5]. Compared to the literature, our group strength can be rated as good.

Participation in the questionnaire was voluntary, just as it was in many other studies [2, 5, 9, 40], which explains the participation rate of the study-group (novice students = 64%, trained students = 39%). This is comparable to the investigation by Lai [5]. The test results did not affect student grades, which probably meant that the questionnaire was not completed, was processed too quickly, or the participant was not focused while they were completing the questionnaire [5]. Due to this, some random result variations might have been introduced [5]. To avoid this, all test results with more than seven unanswered questions were removed from the score [24]. For future comparable studies, it would be beneficial if a reward were provided for the participants.

### Validation

To improve the validation, we should have had the original BQ carried out in addition to the BQD to ensure the criterion validity. This approach was used in the study by Settineri [41].

### Future settings

The achieved values of the BQD reached acceptable data values. Similar to the original Berlin questionnaire, two parts of 15 questions were used. In a future setting, a larger pool of questions including new psychometric valuable items would allow a higher degree of reliability, as already mentioned in the Discussion. The BQD has not been validated in English yet. The questionnaire can currently only be used in German speaking countries. The English version of the BQD must be validated against the German version in the future.

## Conclusions

The Berlin Questionnaire for Dentistry is a reliable and valid test to assess the competence of dental students in EbD. The study highlights the importance of valid measuring instruments to capture Evidence-based Dentistry knowledge. The effort to modify existing questionnaires and instruments for other applications should not be underestimated. Therefore, we hope that this pilot study will encourage further efforts to expand this questionnaire or to develop new instruments that assess EbD skills.

## Supplementary information

**Additional file 1.** Berlin Questionnaire of Dental Competence in Evidence-based Dentistry (German).

**Additional file 2.** Berlin Questionnaire of Dental Competence in Evidence-based Dentistry (translated into English).

## Data Availability

The datasets used and analysed during the current study are available from the corresponding author on reasonable request.

## Abbreviations

ACE: Assessing Competency in EbM
BQ: Berlin Questionnaire
BQD: Berlin Questionnaire Dental
BQM: Berlin Questionnaire Medical
EbD: Evidence-based Dentistry
EbM: Evidence-based Medicine
EbP: Evidence-based Practice
FT: Fresno Test
