# Supplementary material for: Adaptation and validation of the Berlin questionnaire of competence in evidence-based dentistry for dental students: a pilot study
Source: BMC Med Educ. 2020 May 4;20:136. doi: 10.1186/s12909-020-02053-0 (PMC7197120; doi:10.1186/s12909-020-02053-0)
Supplement: Supplementary file 1 — Additional file 1. Berlin Questionnaire of Dental Competence in Evidence-based Dentistry (German). [file 12909_2020_2053_MOESM1_ESM.pdf]

Liebe StudienteilnehmerInnen,
im Rahmen einer Studie möchte ich ein Evaluationsinstrument zu Evidence-based-Dentistry (EbD) untersuchen. Ziel ist es, das zahnmedizinische Studium mit EbD-Inhalten zu optimieren. Das Instrument besteht aus zwei Teilen (Set A und B) mit insgesamt 30 Fragen zu EbD. Ich bitte Sie diese in maximal 45 Minuten zu beantworten.
Herzlichen Dank für Ihre Mitarbeit. ☺

NAME / CODE: ____

(Selbst beliebig wählen: Buchstaben und/ oder Ziffern)

- **Alter: _____ Jahre**
- **Geschlecht:**
  - ❑ weiblich
  - ❑ männlich

- **Vorkenntnisse (Mehrfach-Auswahl)**
  - ❑ Medizinstudium
  - ❑ Zahnmedizinstudium
  - ❑ Zusatzstudium (Public Health, Epidemiologie, etc.)
  - ❑ EBM-Buch gelesen
  - ❑ Bereits an einer EBM-Einführung/-Session (weniger als ein Tag) teilgenommen
  - ❑ Bereits an einem anderen EBM-Kurs (ein Tag oder länger) teilgenommen
  - ❑ Bereits einen EBM-Kurs als Tutor geleitet

- **Selbsteinschätzung EBM-Kenntnisse**
  - ❑ Keine
  - ❑ Wenig
  - ❑ Durchschnitt
  - ❑ Fortgeschritten
  - ❑ Experte

- **Jahre seit Abschluss des ZahnMedizinstudiums**
  _____ Jahre

- **Derzeitiges Haupt-Tätigkeitsfeld (Einfach-Auswahl)**
  - ❑ Direkte Patientenversorgung (Klinik, Praxis)
  - ❑ Gesundheitsverwaltung (einschließlich MDK)
  - ❑ Akademisch (Epidemiologie, Sozialmedizin, Public Health etc.)
  - ❑ Industrie (Pharma etc.)
  - ❑ Medizinische Publizistik (Redaktion, Verlag etc.)
  - ❑ Sonstiges: ______________________________

- **Derzeitige Position**
  - ❑ Studierende/r im ______ Fachsemester
  - ❑ Praktisches Jahr Medizin
  - ❑ Assistenz(zahn)arzt
  - ❑ Assistenz(zahn)arzt mit Facharztbezeichnung
  - ❑ angestellter (Zahn)arzt
  - ❑ angestellter (Zahn)arzt mit Facharztbezeichnung
  - ❑ Oberarzt / Chefarzt
  - ❑ In eigener Praxis
  - ❑ Sonstiges: ______________________________

# - BEGINN SET A -

FRAGE 1

Im Notdienst stellt sich ein Mann mit seit ca. 24h bestehenden Schmerzen im rechten Unterkieferbereich vor. Bei der klinischen Untersuchung ergeben sich keine eindeutigen Zeichen einer Speichelgangentzündung. Sie wissen aber, dass in dieser Altersgruppe trotzdem ungefähr jeder 10. Patient mit diesen Beschwerden eine Speichelgangentzündung ohne typische Zeichen hat.
Sie veranlassen eine Ultraschall-Untersuchung, weil Sie von der letzten internen Qualitätskontrolle wissen, dass der diensthabende Sonographeur bei der Speichelgangentzündungs-Diagnostik gute Werte erreicht hat (Wahrscheinlichkeitsverhältnis [=Likelihood-Ratio] bei positiven Befunden 1,8 ; bei negativen Befunden 0,2).
In diesem Fall legt sich der Sonographeur darauf fest, dass der Patient eine Speichelgangentzündung hat. Als Sie den Chirurgen hinzuziehen wollen, fragt dieser telefonisch, wie hoch Sie jetzt die Wahrscheinlichkeit einschätzen,
dass der Patient tatsächlich eine Speichelgangentzündung hat.

Sie antworten:
A Ungefähr 2%
B Ungefähr 7%
C Ungefähr 15%
D Ungefähr 30%
E Eine Aussage ist vor Eintreffen der Laborbefunde nicht möglich
(Ein unbeschriftetes Fagan-Nomogramm liegt bei)

Antwort 1: _____

FRAGE 2

Der anwesende Praktikant ist von Ihrer Antwort beeindruckt und fragt Sie, wie man ein Wahrscheinlichkeitsverhältnis berechnen kann. Sie erklären ihm, dass sie Ultraschall-Diagnosen aus einem bestimmten Zeitraum mit der tatsächlich richtigen Diagnose (aus der Histologie oder Verlaufsbeobachtung) verglichen wurden. Da Ihnen die Zahlen nicht mehr erinnerlich sind, demonstrieren Sie dies an einem Zahlenbeispiel:

		**Tatsächliche Diagnose Speichelgangentzündung**	
		Ja	Nein
**Sonographische Diagnose**	Ja	90	10
**Speichelgangentzündung**	Nein	20	110

In diesem Beispiel ist das Wahrscheinlichkeitsverhältnis für einen positiven Befund:

A $$0{,}09 = \frac{\frac{10}{(10+110)}}{1 - \frac{20}{(110+90)}}$$

B $$0{,}10 = \frac{\frac{10}{(110+10)}}{\frac{90}{(90+20)}}$$

C $$1{,}54 = \frac{\frac{20}{(20+110)}}{1 - \frac{90}{(90+10)}}$$

D $$9{,}82 = \frac{\frac{90}{(90+20)}}{\frac{10}{(10+110)}}$$

E Das Wahrscheinlichkeitsverhältnis lässt sich aus diesen Angaben nicht berechnen

Antwort 2: _____

FRAGE 3

Der Praktikant ist jetzt völlig begeistert und behauptet über die Patienten in Ihrem Zahlenbeispiel (Frage 2):

1) Ohne jegliche weitere Information könne man feststellen, dass die Beispielpatienten mit pathologischem Ultraschallbefund mit 90-prozentiger Wahrscheinlichkeit tatsächlich eine Speichelgangentzündung haben.

2) Ohne jegliche weitere Information könne man feststellen, dass für die Beispielpatienten mit normalem Ultraschall-Befund die Wahrscheinlichkeit eines Falschbefundes 18% beträgt → [20÷*(20+90)]*

3) Der positive prädiktive Wert berechnet sich direkt aus dem Quotienten der Wahrscheinlichkeitsverhältnisse→ *[90% = 0,09÷0,01]*

Sie sagen ihm:

A Alle Aussagen (1, 2, 3) sind falsch.
B Die erste Aussage (1) ist richtig, die beiden anderen Aussagen (2, 3) sind falsch.
C Die zweite und die dritte Aussage (2, 3) sind richtig, die erste Aussage (1) ist falsch.
D Die erste und die dritte Aussage (1, 3) sind richtig, die zweite Aussage (2) ist falsch.
E Alle Aussagen (1, 2, 3) sind richtig.

Antwort 3: _____

FRAGE 4

Ein Pharmavertreter besucht Sie in Ihrer Praxis und stellt Ihnen ein neues Medikament vor, das in einer großen doppel-blind randomisiert kontrollierten Studie an gesunden Mitarbeitern des Pharmakonzerns eine 50%ige Reduktion des Risikos an einem Speicheldrüsenkarzinom (Mukoepidermoidtumor) zu versterben erreicht hat:

- 4.000 Personen wurden behandelt, von diesen verstarben 4 *(0,1%)* an einem Speicheldrüsenkarzinom.
- Von den 4.000 nicht behandelten Kontroll-Personen verstarben 8 *(0,2%).*

Der Pharmavertreter empfiehlt Ihnen daher, alle Patienten mit dem neuen Medikament zu behandeln. Sie wollen zumindest einem Patienten in dieser Praxis so das Leben retten, stellen aber fest, dass Sie

A dazu 1000 Patienten behandeln müssen *[= 1 ÷ (0,2% - 0,1%)]*
B dazu 2000 Patienten behandeln müssen *[= 8000 ÷ 4]*
C dazu 4000 Patienten behandeln müssen *[= 4 • (1 ÷ 0,1%)]*
D dazu 8000 Patienten behandeln müssen *[= 4000 • 2]*
E die Zahl der Patienten, die Sie dazu behandeln müssen, nicht berechnen können

Antwort 4: _____

FRAGE 5

Sie freuen sich über diese Gelegenheit, der Menschheit zu helfen und rufen einen Kollegen an, um ihm die frohe Botschaft mitzuteilen. Ihr Kollege hat aber bereits die Veröffentlichung der Studiendaten (Frage 4) gelesen und weist Sie auf die Häufung von Lungenödemen unklarer Genese in der Behandlungsgruppe hin:

- 7 Fälle in der Behandlungsgruppe
- 2 Fälle in der Kontrollgruppe.

Sie rechnen nach und stellen fest, dass ein zusätzliches Lungenödem bei

A einer Zahl von 1000 behandelten Patienten *[= 2000 • 0,2%]* zu erwarten ist.
B einer Zahl von 571 behandelten Patienten *[= 4000 ÷ 7]* zu erwarten ist
C einer Zahl von 800 behandelten Patienten *[= 1 ÷ (5 ÷ 4000)]* zu erwarten ist
D einer Zahl von 2000 behandelten Patienten *[= 2 • (1 ÷ 0,1%)]* zu erwarten ist
E einer aus diesen Daten nicht berechenbaren Anzahl von behandelten Patienten zu erwarten ist.

Antwort 5: _____

FRAGE 6

Eine Patientin mit chronischen Zahnschmerzen hat kürzlich in ihrer Fernsehzeitschrift gelesen, dass Formaldehyd-belastete Wohnräume chronische Zahnschmerzen verursachen. Sie möchte, dass die Krankenkasse nun ihren Umzug finanziert. Daher fragt sie, ob der Zusammenhang zwischen chronischen Zahnschmerzen und Formaldehyd in der Raumluft wissenschaftlich bewiesen sei. Sie machen eine entsprechende Literaturrecherche und finden verschiedene Studien zu diesem Thema. *Welches Studien-Design halten sie für die Untersuchung dieser Frage am geeignetsten?*

A Prävalenzstudie
B ökologische Studie
C Fall-Kontroll-Studie
D Prospektive randomisiert-kontrollierte Studie
E Fall-Serie

Antwort 6: _____

FRAGE 7

Im Einzelnen sind bei den von Ihnen gefundenen Studien die folgenden Vorgehensweisen gewählt worden. Welche halten Sie für am besten geeignet, um zu prüfen, ob tatsächlich ein Zusammenhang zwischen Zahnschmerz und Formaldehyd in der Raumluft besteht?

A Bei 100 Patienten mit Zahnschmerzen aus einer Spezialambulanz und 100 Patienten aus Allgemeinzahnarztpraxen ohne Zahnschmerz, die von Alter, Geschlecht und Einkommen den Zahnschmerzpatienten entsprechen, wird der Formaldehydgehalt in den Wohnräumen gemessen.

- **Vergleich des mittleren Formaldehydgehaltes in beiden Gruppen.**

B Befragung von 500 Patienten einer Ambulanz für Umweltmedizin, ob sie 1. Zahnschmerzen haben und 2. meinen, dass ihre Wohnräume formaldehydbelastet sind.

- **Vergleich der Häufigkeiten der Antwort zu 2. (Formaldehydbelastete Wohnräume) bei Patienten mit Zahnschmerzen vs. ohne Zahnschmerzen**

C Befragung von Mietern einer Wohnungsbaugesellschaft mit einem Zahnschmerzfragebogen. Gleichzeitige Durchführung eines Haut-Tests auf Formaldehydallergie.

- **Vergleich der Häufigkeit von Zahnschmerzen bei Mietern mit Formaldehydallergie vs. übrige Mieter.**

D Zweimalige Messung der Formaldehydkonzentration im Blut von Zahnschmerzpatienten aus einer Schmerzambulanz im Abstand von einem Jahr.

- **Vergleich der Werte zu Beginn mit denen am Ende der Beobachtung.**

E Befragung von Personen, die neu in vermutlich formaldehydbelastete Wohnräume einer städtischen Wohnungsbaugesellschaft ziehen und von langjährigen Mietern solcher Wohnungen mit einem Zahnschmerzfragebogen.

- **Vergleich der Häufigkeit von Zahnschmerzen zwischen neuen und langjährigen Mietern.**

Antwort 7: _____

FRAGE 8

In einer großen Studie wurde untersucht, ob eine neue antibakterielle Mundspülung die Mortalität durch bakterielle Endokarditis senkt.
An der Studie nahmen 1000 Risikopatienten teil. Nach dem Zufallsprinzip wurden 500 Patienten in die Behandlungsgruppe und 500 in die Kontrollgruppe eingeteilt. Die Behandlungsgruppe erhielt über ein Jahr die antibakterielle Mundspülung, die Kontrollgruppe eine genauso aussehende Mundspülung (als Placebo). Die Patienten der Behandlungsgruppe wurden monatlich auf das Auftreten von Nebenwirkungen untersucht, die Patienten der Kontrollgruppe hingegen nur halbjährlich.
In der Behandlungsgruppe starben 10 Patienten weniger an bakterieller Endokarditis als in der Kontrollgruppe *($p<0,01$).* Sie überlegen, ob die Qualität der Studie Sie überzeugt.

Folgende Feststellung hierzu trifft am ehesten zu:

A Die Studie wurde nicht prospektiv durchgeführt
B Die Studie wurde nicht randomisiert durchgeführt
C Die Studie wurde nicht doppelblind durchgeführt
D In der Studie wurde nicht der für die Fragestellung wirklich interessierende Endpunkt, sondern ein Surrogatendpunkt untersucht.
E In dieser prospektiven randomisiert-kontrolliert doppelblind durchgeführten Studie wurde der für die Fragestellung interessierende Endpunkt untersucht.

Antwort 8: _____

FRAGE 9

1) Aus den vorhandenen Angaben (Frage 8) kann man errechnen, wie viele Patienten wie in der Studie mit der antibakteriellen Mundspülung behandelt werden müssen, um einen zusätzlichen Endokarditis-Todesfall zu verhindern.

2) Für die Berechnung der Zahl der Patienten, die behandelt werden müssen, um einen zusätzlichen Todesfall zu verhindern, muss man die relative Verringerung des Sterbe-Risikos der Behandlungsgruppe gegenüber der Kontrollgruppe kennen.

3) In den vorhandenen Angaben (Frage 8) ist die relative Verringerung des Sterbe-risikos der Behandlungsgruppe gegenüber der Kontrollgruppe (relative Risikoreduktion) angegeben.

A Alle Aussagen (1, 2, 3) sind falsch.
B Die erste Aussage (1) ist richtig, die beiden anderen Aussagen (2, 3) sind falsch.
C Die zweite und die dritte Aussage (2, 3) sind richtig, die erste Aussage (1) ist falsch.
D Die erste und die dritte Aussage (1, 3) sind richtig, die zweite Aussage (2) ist falsch.
E Alle Aussagen (1, 2, 3) sind richtig.

Antwort 9:_____

FRAGE 10

Sie sind Fachärztin für Mund-, Kiefer-, Gesichtschirurgie. Ihre 63jährige Patientin hat als Zufallsbefund eine 70%ige Stenose der linken A. facialis. Sie fragen sich, ob dies eine Indikation zur Überweisung zur Endarteriektomie darstellt und finden eine Studie, die bei asymptomatischen Patienten mit 70%iger Stenose (vergleichbar Ihrer Patientin) bei 5 Jahren Nachbeobachtung keinen Nutzen durch eine Operation gegenüber konservativer Behandlung findet. In einer Untergruppenanalyse (insgesamt 13 Untergruppen) zeigt sich nach Berücksichtigung des Risikofaktorstatus zu Studienbeginn, dass nur diejenigen Frauen, die das erste Jahr ohne Insult oder TIA überstanden haben, im Verlauf der nachfolgenden 4 Jahre ein statistisch signifikanten Nutzen ($p < 0.03$) von der Endarteriektomie hatten.

Welche Aussage trifft zu? :

A Das signifikante Ergebnis belegt den Operationsnutzen bei Frauen und reicht allein aus, um die Operationsindikation zu rechtfertigen.

B In der Untergruppen-Analyse wurde für andere Risikofaktoren korrigiert, was meistens zu irreführenden Schlussfolgerungen führt.

C Mit Untergruppen-Analysen lässt sich die Ausbeute an verlässlichen Ergebnissen aus randomisiert kontrollierten Studien maximieren.

D Mit zunehmender Zahl von nachträglich gebildeten Untergruppen steigt die Gefahr, dass in einer Untergruppe irrtümlich ein Nutzen gefunden wird, der in Wirklichkeit nicht existiert.

E Die Untergruppenanalyse beweist in diesem Fall, dass asymptomatische A.Facialis-Stenosen für Frauen gefährlicher sind als für Männer.

Antwort 10:**_____**

FRAGE 11

In Fortbildung über die Auswirkung von Medikamenten nach Mundbodenkarzinomen werden für unterschiedliche Medikamente folgende randomisierte kontrollierte Studien vorgestellt, die alle bei Patienten mittleren Alters (Alter: 55 J.) mit normaler Mundhygiene und Tabakgenuss über einen Zeitraum von fünf Jahren gegen ein Placebo getestet wurden.

1) In der bolivianischen Studie senkte Therapie II das Risiko für einen tödlichen Ausgang um25%.

2 In einer argentinischen Studie senkte die Therapie I das Risiko tödlicher Ausgänge um 30%.

3) In einer chilenischen Studie verstarben in der Gruppe mit Behandlung III 3% der Patienten und in der Kontrollgruppe 4% der Patienten an einem Mundbodenkarzinom.

Welche Aussage trifft zu?

A Therapie 1 ist zu bevorzugen da diese Therapie das Todesfall-Risiko am stärksten senkt.

B Therapie 3 ist zu bevorzugen, da bei ihr der Anteil der Patienten die von der Behandlung profitieren am größten ist.

C Bei Therapie 2 und 3 ist die relative Reduktion des Risikos eines Todesfalles gleich groß.

D Bei Therapie 1 und 3 ist die relative Reduktion des Risikos eines tödlichen Ausganges gleich groß.

E Für keine der Therapien wird das Risiko unbehandelter Patienten (Control-Event-Rate) angegeben.

Antwort 11:**_____**

<u>FRAGE 12</u>

In einer kieferchirurgischen Poliklinik besteht eine Prävalenz von 30% für Plattenepithelkarzinome. 1000 aufeinanderfolgende Patienten werden in eine Studie für einen neuen, nicht invasiven, wenig belastenden diagnostischen Test zum Nachweis von Plattenepithel-Karzinomen aufgenommen. Mit Hilfe des Tests werden 630 Patienten als richtig negativ erkannt, d.h. richtigerweise tumorfrei. Die Anzahl an falsch negativen und falsch positiven Patienten ist identisch.

	**Goldstandard positiv**	**Goldstandard negativ**	
**Test positiv**			
**Test negativ**			

(4-Felder-Tafel für die eigene Berechnung des Ergebnisses)

<u>Welche 4-Felder-Tafel entspricht diesen Angaben?</u>

4-Felder-Tafel A

	Goldstandard positiv	*Goldstandard negativ*	
Test positiv	230	70	300
Test negativ	70	630	700
	300	700	1000 Patienten

4-Felder-Tafel B

	Goldstandard positiv	*Goldstandard negativ*	
Test positiv	300	0	300
Test negativ	0	700	700
	300	700	1000 Patienten

4-Felder-Tafel C

	Goldstandard positiv	*Goldstandard negativ*	
Test positiv	270	100	370
Test negativ	30	600	630
	300	700	1000 Patienten

4-Felder-Tafel D

	Goldstandard positiv	*Goldstandard negativ*	
Test positiv	670	30	700
Test negativ	30	270	300
	700	300	1000 Patienten

4-Felder-Tafel E

	Goldstandard positiv	*Goldstandard negativ*	
Test positiv	300	70	370
Test negativ	70	560	630
	370	630	1000 Patienten

Antwort 12:_____

FRAGE 13

Vor einiger Zeit hatten Sie eine 40 jährige Patientin wegen eines getasteten Knotens im Vestibulum zum MKG-Chirurgen überwiesen. Der Knoten wurde nach Punktion als gutartig eingestuft. Die Patientin bringt den Befund mit und möchte von Ihnen wissen, ob die Zyste bedeutet, dass sie besonders gefährdet ist, Krebs zu entwickeln. Sie finden dazu mehrere Studien. Welche Studie ist am ehesten geeignet die prognostische Bedeutung von gutartigen Zysten bei Patienten aus einer Normalbevölkerung zu beurteilen?

A Studie aus MKG-chirurgischen Ambulanz einer Pariser Universitätsklinik: Von 1996 - 1998 wurden alle Patienten mit Karzinom nach Vorbefunden von tastbaren Zysten befragt. Zum Vergleich wurden Patienten ohne Karzinom auf tastbare Zysten untersucht. Patienten mit Karzinom hatten 20% weniger Zysten, als Patienten ohne Karzinom.

B Studie aus einem Universitätsklinikum in Boston: Die Röntgenbilder von 1500 Patienten mit Karzinom aus den letzten 10 Jahren wurden nachträglich auf das Vorhandensein von Zysten untersucht. Dabei fanden sich bei 30% der Patienten auch größere Zysten.

C Studie aus der Ambulanz des einzigen Überweisungszentrums in Ostschottland für Patienten mit Problemen im Vestibulum: Alle Patienten, die wegen tastbarer Zysten abgeklärt worden waren, wurden 10 Jahre später nachuntersucht bzw. im Krebsregister und Sterberegister gesucht. Verglichen mit der Normalbevölkerung war die Häufigkeit für Karzinome doppelt so hoch.

D Studie aus der Pathologie einer Spezialklinik im Ruhrgebiet: Alle Histologien von Gewebe aus den letzten 10 Jahren wurde erfasst. Bei 11% der Patienten, bei denen eine gutartige Zyste diagnostiziert wurde, wurde innerhalb des Erfassungszeitraums auch ein Karzinom histologisch erfasst.

E Multizentrische Studie in mehreren Kreiskrankenhäusern: Erfahrene Chirurgen wurden Befragt, wie oft nach ihrer Erfahrung bei Karzinom- Patienten in der Vorgeschichte Gutartige Zysten diagnostiziert wurden. Der Mittelwert der Angaben lag bei 37%.

Antwort 13:**_____**

FRAGE 14

In den deutschsprachigen Ländern wird der frei flottierende Thrombus bei einer tiefen Venenthrombose wegen der mutmaßlich höheren Emboliegefahr besonders gefürchtet. Sie möchten *aus persönlichem Interesse* wissen, ob Patienten mit einem frei flottierenden Thrombus ein höheres Risiko für eine Lungenembolie haben als Patienten mit wandständigem Thrombus.

1) Dies ist eine Frage über Prognose
2) Dies ist eine Frage über Nebenwirkungen
3) Diese Frage wird am besten in einer Fall-Kontrollstudie untersucht
4) Diese Frage wird am besten in einer Kohortenstudie untersucht.
5) Diese Frage wird am besten in einer randomisierten kontrollierten Studie untersucht

Welche Aussage trifft zu?

A 1 und 3 sind richtig
B 1 und 4 sind richtig
C 1 und 5 sind richtig
D 2 und 4 sind richtig
E 2 und 5 sind richtig

Antwort 14:_____

FRAGE 15:

Die Meta-Analyse ist ein Studientyp, der in letzter Zeit zunehmende Bedeutung erlangt hat. Welche Aussage trifft zu?

A Durch die Technik der Meta-Analyse ist die Durchführung großer Studien mit vielen Patienten (Mega-Trials) weniger bedeutsam geworden.

B In systematischen Untersuchungen konnte nachgewiesen werden, dass Studien, die in der jeweiligen Landessprache veröffentlich wurden, schlechter sind, als die Studien in englischen Zeitschriften. Aus diesem Grunde genügt für eine Meta-Analyse die Berück-sichtigung der englischsprachigen Literatur.

C Studien mit größeren Patientenzahlen finden häufig auch einen größeren Therapie- Effekt als Studien mit einer kleineren Patientenzahl.

D In einer Meta-Analyse randomisierter Studien, die insgesamt eine hohe Patientenzahl Umfasst, kann die Wirksamkeit von Maßnamen genauer geschätzt werden, als in Kleineren Einzelstudien.

E Bei der Meta-Analyse können Unterschiede in der Fragestellung oder den Patientenpopulationen der einzelnen Studien mit Hilfe der Statistik ausgeglichen werden.

Antwort 15:_____

- ENDE SET A -

# - BEGINN SET B -

FRAGE 1

Sie sind Zahnarzt auf der MKG-chirurgischen Station eines Krankenhauses auf dem Land (Grundversorgung). Einer 46-jährigen Patientin wurde in dieser Woche ein kleines Mundbodenkarzinom (pT1 pN0 M0) operativ entfernt. Die Patientin ist aufgrund der erst kürzlich mitgeteilten Diagnose völlig aufgelöst und möchte nun von Ihnen wissen, wie lange sie ohne weitere Therapiemaßnahmen noch zu leben hat. Sie sind sich nicht sicher, ob und in welchem Maße die Lebenserwartung der Patientin nach erfolgreicher OP ohne Nachbehandlung eingeschränkt ist. Sie wollen die Frage anhand geeigneter Studien beantworten.

Welcher Studientyp eignet sich am besten zur Beantwortung dieser Frage?
A Fall-Kontroll-Studie
B Querschnittsstudie
C Kohortenstudie
D Fall-Serie
E Prävalenzstudie

Antwort 1:_____

FRAGE 2

Bei Ihrer Literaturrecherche stoßen Sie auf folgende Studien. Welche ist am besten zur Beantwortung der Frage der Patientin (s. Frage 1) geeignet?

A In einer großen Multi-Center-Studie wurden 3600 Frauen nach operativer Entfernung eines Mundbodenkarzinoms in zwei Gruppen randomisiert. Eine Gruppe wurde nachbestrahlt, die andere erhält eine adjuvante Chemotherapie. Die Überlebensraten beider Gruppen werden angegeben.

B In einer süddeutschen Kleinstadt wurden alle Frauen über 40 befragt, ob sie schon eine Mundschleimhaut-OP hinter sich haben. Für die Gruppe der Frauen mit Mundbodenkarzinom-OP in der Anamnese wird aus der Altersverteilung die Sterberate für die verschiedenen Tumorstadien kalkuliert.

C Der mittlerweile pensionierte Leiter Ihrer Abteilung hat über Jahre Fälle von Frauen mit T1-Karzinomen, die er operiert hat, gesammelt und seine Erfahrungen wurden zu seinem 65-sten in einem Sonderband publiziert. Keine einzige Patientin ist vorzeitig gestorben.

D In einem deutschen Stadtstaat haben sich alle MKG-chirurgischen-Kliniken zu einer Studiengruppe zusammengeschlossen. Alle Frauen, bei denen operativ ein Mundbodenkarzinom entfernt wurde, werden mit ihrem Tumorstadium zum Zeitpunkt der OP registriert. In jährlichen Abständen wird überprüft, ob die Frauen noch am Leben sind. Die Überlebensrate wird nach Tumorstadium gruppiert angegeben.

E Das pathologische Institut einer renommierten deutschen Universitätsklinik hat alle Sektionsfälle, bei denen in der Vorgeschichte ein Mundbodenkarzinom operiert wurde registriert. Aus der Differenz zwischen OP-Datum und Todesdatum wird die Überlebensrate berechnet und gruppiert nach Tumorstadien angegeben.

Antwort 2:_____

FRAGE 3

Die Patientin hat in einer (amerikanischen) Frauen-Zeitschrift eine Anzeige für Immuntherapie gelesen. Sie möchte von Ihnen wissen, ob es sich bei der Immuntherapie um Medikamente mit einer nachgewiesenen Wirkung handelt.

1) Für den Nachweis einer Medikamentenwirkung ist eine doppel-blinde randomisierte kontrollierte Studie ein geeigneter Studien-Typ.
2) Die Randomisierung sollte zu einer ungefähr gleichen Verteilung der Patienteneigenschaften zwischen Behandlungs- und Kontrollgruppe führen.
3) Durch die Randomisierung sollten auch unbekannte Faktoren, die für die Heilung bedeutsam sein können, in beiden Gruppen mit gleicher Wahrscheinlichkeit wirksam werden.

A Alle Aussagen (1, 2, 3) sind falsch.
B Die erste Aussage (1) ist richtig, die beiden anderen Aussagen (2, 3) sind falsch.
C Die zweite und die dritte Aussage (2, 3) sind richtig, die erste Aussage (1) ist falsch.
D Die erste und die dritte Aussage (1, 3) sind richtig, die zweite Aussage (2) ist falsch.
E Alle Aussagen (1, 2, 3) sind richtig.

Antwort 3:_____

FRAGE 4

In einer Studie zur Wirksamkeit von Immuntherapie bei Plattenepithelkarzinomen (PEC) wurden in 37 amerikanischen Kliniken insgesamt 10.000 Frauen direkt nach operativer Entfernung eines PEC eingeschlossen. In jede Klinik wurde eine Liste von mit einem Zufallsgenerator erzeugten Zahlen ausgehängt. Bei Einschluss einer Patientin wurde die nächste noch nicht benutze Zahl der Liste dieser Patientin zugeordnet und dann durchgestrichen. Handelte es sich um eine gerade Zahl wurde die Patientin für drei Jahre mit 2x30 mg der Immuntherapie täglich behandelt. Bei einer ungeraden Zahl wurde die entsprechende Patientin über den gleichen Zeitraum beobachtet aber nicht behandelt. Am Ende des Beobachtungszeitraums von drei Jahren wurde festgestellt, wie viele Patientinnen in der behandelten und in der nicht behandelten Gruppe jeweils verstorben waren. In der behandelten Gruppe verstarben innerhalb von drei Jahren 800 von 5000 Patientinnen, in der nicht behandelten Gruppe 1000 von 5000.

Die Studie hatte folgende Eigenschaften
1) randomisiert
2) verdeckt randomisiert (concealed allocation)
3) doppel-blind
4) kontrolliert
5) multi-zentrisch

Welche Eigenschaften hat die Studie?
A 1, 2 und 5 sind richtig
B 1, 3 und 5 sind richtig
C 1, 4 und 5 sind richtig
D 2 und 3 sind richtig
E 4 und 5 sind richtig

Antwort 4:_____

FRAGE 5

Welche Aussage(n) zur Beeinflussung des Sterblichkeitsrisikos durch die Behandlung trifft in dieser Studie zu?

1) In der behandelten Gruppe sank das Risiko zu versterben um 20% des Risikos der nicht behandelten Gruppe ***[relative Risikoreduktion 20% = (1000-800)÷1000]***

2) In der behandelten Gruppe sank das Risiko zu versterben um 80% des Risikos der nicht behandelten Gruppe ***[relative Risikoreduktion 80% = (800÷1000)]***

3) In der behandelten Gruppe lag das Sterblichkeitsrisiko um 2 Prozentpunkte unter dem der nicht behandelt.Gruppe ***[absolute Risikoredukt. 2%= (1000-800)÷10.000]***

4) In der behandelten Gruppe lag das Sterblichkeitsrisiko um 4 Prozentpunkte unter dem der nicht behandelten Gruppe ***[absolute Risikoredukt.4% = (1000-800) ÷5.000]***

5) In der behandelten Gruppe lag das Sterblichkeitsrisiko um 10 Prozentpunkte unter dem der nicht behandelten Gruppe ***[absolute Risikoreduktion 10% =1000÷10.000]***

A 1 und 3 sind richtig
B 1 und 4 sind richtig
C 1 und 5 sind richtig
D 2 und 4 sind richtig
E 2 und 5 sind richtig

Antwort 5:_____

FRAGE 6

Angenommen in dieser Studie (Frage 5) habe die relative Risikoreduktion 50% und die absolute Risikoreduktion 2% betragen.
Wie viele Patientinnen müssten dann mit der Immuntherapie behandelt werden, um einen zusätzlichen Todesfall zu verhindern?

A *48 = 50 – 2*
B *50 = 1 ÷ 2%*
C *52 = 50 + 2*
D *100 = 5000 • 2%*
E Für diese Berechnung sind weitere Angaben notwendig

Antwort 6:_____

FRAGE 7

In einer großen multi-zentrischen doppel-blinden Studie wurde untersucht, ob durch Dauermedikation mit einem Antiallergikum die Gefahr von anaphylaktischen Reaktionen gesenkt werden kann. Behandlungs- und Placebogruppe waren genau gleich groß.
In der Placebo-Gruppe erlitten 25% der Patienten eine anaphylaktische Reaktion.
Die absolute Risiko-Reduktion betrug 2,4% (95% Konfidenzintervall = 0,5% - 20%)
Bitte prüfen Sie folgende Feststellungen

1) Durch die Einnahme des Antiallergikums (Behandlung) wurde das Risiko eine anaphylaktische Rektion zu erleiden um etwa 10% reduziert.
2) Durch die Einnahme des Antiallergikums (Behandlung) wurde bei etwa 2,4% der Patienten eine anaphylaktische Reaktion verhindert
3) Der Unterschied zwischen Placebo- und Antiallergikum-(Behandlungs-)Gruppe ist statistisch signifikant

Welche Aussage(n) treffen zu?

A keine Aussage ist richtig
B 1 ist richtig
C 2 ist richtig
D 2 und 3 sind richtig
E Alle Aussagen sind richtig

Antwort 7:_____

FRAGE 8

In der Notaufnahme Ihres Krankenhauses soll ein neuer Test für den Nachweis von Dysplasien in Mundschleimhautveränderungen eingeführt werden. Die Bürstenbiopsie hat nur zwei mögliche Ergebnisse: Positiv (=Dysplasie) oder Negativ (=Keine Dysplasie). Die Sensitivität beträgt 99%, die Spezifität liegt bei 60%.

1) Ein diagnostischer Test mit einer sehr hohen Sensitivität ist gut für den Ausschluss der untersuchten Erkrankung (hier: Dysplasie) geeignet

2) Eine hohe Sensitivität bedeutet, dass der diagnostische Test die gesuchte Erkrankung mit hoher Wahrscheinlichkeit findet, wenn sie tatsächlich vorhanden ist.

3) Bei einem diagnostischen Test mit einer sehr hohen Sensitivität ist die Wahrscheinlichkeit sehr niedrig, dass trotz eines negativen Test-Ergebnisses (hier:Keine Dysplasie) die gesuchte Erkrankung (hier: Dysplasie) trotzdem vorliegt

A Alle Aussagen (1, 2, 3) sind falsch.
B Die erste Aussage (1) ist richtig, die beiden anderen Aussagen (2, 3) sind falsch.
C Die zweite und die dritte Aussage (2, 3) sind richtig, die erste Aussage (1) ist falsch.
D Die erste und die dritte Aussage (1, 3) sind richtig, die zweite Aussage (2) ist falsch.
E Alle Aussagen (1, 2, 3) sind richtig.

Antwort 8:_____

FRAGE 9

Die Eigenschaften der Bürstenbiopsie wurden in einer Studie untersucht. Es wurden bei einer Prävalenz von 30% für Dysplasien 1000 aufeinanderfolgende Patienten in die Studie aufgenommen. Mit Hilfe des Tests werden 630 Patienten als richtig negativ erkannt, d.h. richtigerweise dysplasiefrei. Die Anzahl an falsch negativen und falsch positiven Patienten ist identisch.

	**Goldstandard positiv**	**Goldstandard negativ**	
**Test positiv**			
**Test negativ**			

(4-Felder-Tafel für die eigene Berechnung des Ergebnisses)

Welche 4-Felder-Tafel entspricht diesen Angaben?

4-Felder-Tafel A

	Goldstandard positiv	*Goldstandard negativ*	
Test positiv	230	70	300
Test negativ	70	630	700
	300	700	1000 Patienten

4-Felder-Tafel B

	Goldstandard positiv	*Goldstandard negativ*	
Test positiv	300	0	300
Test negativ	0	700	700
	300	700	1000 Patienten

4-Felder-Tafel C

	Goldstandard positiv	*Goldstandard negativ*	
Test positiv	270	100	370
Test negativ	30	600	630
	300	700	1000 Patienten

4-Felder-Tafel D

	Goldstandard positiv	*Goldstandard negativ*	
Test positiv	670	30	700
Test negativ	30	270	300
	700	300	1000 Patienten

4-Felder-Tafel E

	Goldstandard positiv	*Goldstandard negativ*	
Test positiv	300	70	370
Test negativ	70	560	630
	370	630	1000 Patienten

***Antwort 9:*_____**

FRAGE 10

In einer anderen Studie wurde die Eignung des Tests zur Diagnose bzw. Ausschluss eines Lichen planus untersucht. Bei Untersuchung von 800 Patienten mit und ohne Lichen ergaben sich folgende Ergebnisse:

		**Tatsächliche Diagnose Lichen**	
		Ja	Nein
**Chairside-Test-Ergebnis**	Lichen	90	210
	Keine Lichen	110	190

Bei diesem Test beträgt die Wahrscheinlichkeit, dass ein Patient, über den Sie sonst nichts wissen, tatsächlich einen Lichen hat, wenn der Test positiv („Lichen“) ausfällt (Positiver prädiktiver Wert):

A *50% = (90+110) ÷ (210+190)*
B *30% = 90÷(90+210)*
C *45% = 90÷(90+110)*
D *100% = (110+190) ÷(90+210)*
E *27,5% = 110÷(210+190)*

Antwort 10:_____

FRAGE 11

Sie benutzen den Test nun zum ersten Mal selbst in der Notaufnahme bei einem älteren Mann. Sie sind sich überhaupt nicht im Klaren, ob er einen Lichen hat oder nicht. Sie schätzen daher aufgrund Ihres klinischen Eindrucks die Wahrscheinlichkeit, dass ein Lichen vorliegt, auf 50%. Der daraufhin durchgeführte Bürstenbiopsie- Test ist positiv. Sie wissen aus der oben angeführten Studie, dass das Wahrscheinlichkeitsverhältnis für einen positiven Befund 7,7 und für einen negativen Befund 0,3 beträgt. Daher schätzen Sie nun die Wahrscheinlichkeit, dass eine Lichen vorliegt, auf:

A Ungefähr 90%
B Ungefähr 75%
C Ungefähr 60%
D Ungefähr 45%
E Eine Aussage ist vor Durchführung einer Exzisionsbiopsie nicht möglich
(Ein unbeschriftetes Fagan-Nomogramm liegt bei)

Antwort 11:_____

FRAGE 12

Der Laborleiter bietet an, dass die Dysplasie-Bestimmung auch mit einer genaueren Histologie in seinem Labor erfolgen kann. Denn im Labor kann die Anzahl der dysplastischen Zellen genau bestimmt und als Zahlenwert angegeben werden. Dadurch soll die Aussagekraft größer sein, als bei dem Chairside-Test, der nur Positiv oder Negativ als Ergebnis liefert. Der Laborleiter unterstreicht seine Argumentation mit einer Studie, in der die Zahl der dysplastischen Zellen in fünf Stufen eingeteilt wurde.
Für insgesamt 2579 Patienten mit und ohne Dysplasien ergaben sich folgende Ergebnisse:

**Ergebnisstufe**	**Dysplastische Zellen**	**Pat. mit Lichen**	**Pat. Ohne Lichen**
Hoch positiv	> 55/dl	474 (59%)	20 (1,1%)
Mäßig positiv	40 – 55/dl	175 (22%)	79 (4,5%)
Neutral	25 – 39/dl	82 (10%)	171 (10%)
Mäßig negativ	10 – 24/dl	30 (3,7%)	168 (9,5%)
Stark negativ	< 10/dl	48 (5,9%)	1332 (75%)
	Gesamt:	809 (100%)	1770 (100%)

Das Wahrscheinlichkeitsverhältnis (=Likelihood-Ratio) für ein mäßig positives Ergebnis (40 – 55/dl) ist in dieser Studie:

A $4{,}8 = \frac{\frac{175}{809}}{\frac{79}{1770}}$

B $1{,}0 = \frac{\frac{175}{79}}{\frac{1770}{809}}$

C $0{,}02 = \frac{\frac{20}{474}}{\frac{175}{79}}$

D $0{,}01 = \frac{\frac{30+48}{168+1332}}{\frac{474+175}{20+79}}$

E Das Wahrscheinlichkeitsverhältnis lässt sich mit diesen Angaben nicht berechnen

Antwort 12:_____

FRAGE 13

Die Meta-Analyse ist eine wichtige Methode zur Zusammenfassung verschiedener Studien. Welche Eigenschaften verringern in den meisten Fällen die Glaubwürdigkeit einer Meta-Analyse?

1) Getrennte Auswertung von Studien mit signifikantem und nicht-signifikantem Ergebnis
2) Getrennte Auswertung von randomisierten und nicht-randomisierten Studien
3) Beschränkung auf randomisierte Studien
4) Beschränkung auf englisch-sprachige Publikationen
5) Beschränkung auf Publikationen zu nur einem von verschiedenen denkbaren therapeutischen Vorgehen

Die Glaubwürdigkeit einer Meta-Analyse wird durch folgende Eigenschaften meist verringert:

A 2
B 1 und 4
C 2 und 4
D 1, 2, 4 und 5
E alle

Antwort 13:_____

FRAGE 14

Die Benutzung von Funktelefonen wird immer wieder als Gesundheitsrisiko diskutiert. In der MKG-Chirurgie Ihres Hauses wurden in kurzer Zeit mehrere Fälle des extrem seltenen (weltweit jährliche Inzidenz unter 1/100.000) extrem bösartigen Ohrmuschelkrebses (kurz: EBOK) bei Handy-Benutzern diagnostiziert. Der
Chefarzt fragt Sie daraufhin, ob wissenschaftlich belegt sei, dass Handy-Benutzung EBOK verursacht. Sie starten umgehend eine Literatur-Recherche, um diese Frage zu beantworten. Welches Studien-Design halten Sie für am besten zur Untersuchung dieser Frage geeignet?

A Prävalenzstudie
B ökologische Studie
C Fall-Kontroll-Studie
D Prospektive randomisiert-kontrollierte Studie
E Fall-Serie

Antwort 14:_____

FRAGE 15

Sie finden bei Ihrer Literatur-Recherche folgende Studien. Welche ist am besten zur Beantwortung der Frage geeignet?

A In Frankreich wurden Fälle von EBOK per Zeitungsannonce und Kontaktierung von Fachärzten gesucht. Jedem EBOK-Patienten wurden vier Patienten aus der Praxis des behandelnden Arztes zugeordnet, die in Geschlecht, Alter und Einkommen mit dem EBOK-Patienten übereinstimmen, aber nicht an EBOK erkrankt sind. Der Anteil an Handy-Benutzern (aktuell und früher) bei EBOK-Patienten und Nicht-EBOK Patienten wurde verglichen.

B In einer burmesischen Provinzhauptstadt wurde die Verwaltung (240 Personen) mit Handys ausgerüstet. Handys sind ansonsten in Myanmar (=Burma) strengstens verboten. Nach 5 Jahren wurden alle Verwaltungsangestellten auf EBOK untersucht. Die Häufigkeit von EBOK bei diesen Personen wurde mit der Häufigkeit von EBOK bei normalen Einwohnern der Provinz verglichen.

C In einem Krebsregister in Ostdeutschland werden alle Krebs-Todesfälle erfasst. Die Veränderung der Zahl von EBOK-Fällen im Zeitraum 1980 bis 1989 zum Zeitraum 1990 bis 1999 wurde miteinander verglichen.

D In Italien wurde im Jahr 1996 die Inzidenz von EBOK-Fällen in Nord- und Süditalien erhoben. Der Unterschied in der Inzidenz zwischen beiden Regionen wurde mit dem Unterschied in den Pro-Kopf-Handy-Gesprächsminuten zwischen beiden Regionen verglichen.

E In einer Befragung der Mehrzahl der Chefärzte deutscher MKG-Kliniken wurde erhoben, zu welchem Anteil nach Meinung der Befragten bei ihren Patienten EBOK auf die Benutzung eines Handys zurückzuführen war.

Antwort 15: ____

- ENDE SET B -
