# Supplementary material for: Adaptation and validation of the Berlin questionnaire of competence in evidence-based dentistry for dental students: a pilot study
Source: BMC Med Educ. 2020 May 4;20:136. doi: 10.1186/s12909-020-02053-0 (PMC7197120; doi:10.1186/s12909-020-02053-0)
Supplement: Supplementary file 2 — Additional file 2. Berlin Questionnaire of Dental Competence in Evidence-based Dentistry (translated into English). [file 12909_2020_2053_MOESM2_ESM.pdf]

*Dear study participants,*
*As part of a study, I would like to examine an evaluation tool for Evidence-based Dentistry (EbD). The goal is to optimise dental studies with EbD content. The instrument consists of two parts (part A and B) with a total of 30 questions about EbD. Please answer these within a maximum of 45 minutes.*
*Thank you for your cooperation. ☺*

***NAME / CODE:*** ________________________________________________
(Choose: letters and/or numbers)

- **Age: _____ years**
- **Gender:**
  - ❑ female
  - ❑ male

- **Previous knowledge (multiple choice)**
  - ❑ Study of medicine
  - ❑ Study of dental medicine
  - ❑ Additional studies (public health, epidemiology, etc.)
  - ❑ Already read an EbM book
  - ❑ Already participated in an EbM introduction / session (shorter than a day)
  - ❑ Already participated in another EbM course (one day or longer)
  - ❑ Already led an EbM course as a tutor

- **Self-assessment EbM knowledge**
  - ❑ None
  - ❑ Little
  - ❑ Average
  - ❑ Advanced
  - ❑ Expert

- **Years since graduation**
  _____ years

- **Current main field of activity (single- choice)**
  - ❑ Direct patient care (clinic, practice)
  - ❑ Health administration (including MDK)
  - ❑ Academic (epidemiology, social medicine, public health etc.)
  - ❑ Industry (pharmaceuticals, etc.)
  - ❑ Medical journalism (editors, publishers, etc.)
  - ❑ Other: ______________________________

- **Current position**
  - ❑ Student in the ______ semester
  - ❑ Practical year medicine
  - ❑ Assistant doctor/ dentist
  - ❑ Assistant doctor/ dentist as a specialist
  - ❑ Employee (dental) doctor
  - ❑ Employee (dental) doctor as a specialist
  - ❑ Senior doctor/ chief physician
  - ❑ In own medical office
  - ❑ Other: ______________________________

# - BEGINNING PART A -

## QUESTION 1

A man arrives in the emergency service with pain in his right lower jaw area that has been present for about 24 hours. There are no clear signs of salivary gland inflammation on clinical examination. However, you know that in the patient's age group, approximately every tenth patient with these symptoms has salivary gland inflammation without typical signs.
You initiate an ultrasound examination because you know from the last internal quality control that the sonographer on duty has achieved good results in salivary gland diagnostics (probability ratio [= likelihood ratio] for positive findings 1.8, for negative results 0.2).
In this case, the sonographer determines that the patient has inflammation of the salivary gland. When you telephone the surgeon, he asks you what the likelihood is that the patient actually has inflammation of a salivary gland.

*You answer:*
**A** About 2%
**B** About 7%
**C** About 15%
**D** About 30%
**E** A statement is not possible before the laboratory findings arrive (An unlabelled Fagan nomogram is included)

***Answer 1:*** _____

## QUESTION 2

The trainee who is present is impressed with your answer and asks you how to calculate a probability ratio. You tell him that the ultrasound diagnoses from a specific period of time are compared to the actual correct diagnoses (from histology or follow-up). Since you do not remember the numbers, demonstrate this using a numerical example:

| | | **Actual diagnosis of salivary gland inflammation** | |
|---|---|---|---|
| | | Yes | No |
| **Sonographic diagnosis** | Yes | 90 | 10 |
| **Salivary gland inflammation** | No | 20 | 110 |

In this example, the probability ratio for a positive finding is:

**A** $0{,}09 = \dfrac{\frac{10}{(10 + 110)}}{1 - \frac{20}{(110 + 90)}}$

**B** $0{,}10 = \dfrac{\frac{10}{(110 + 10)}}{\frac{90}{(90 + 20)}}$

**C** $1{,}54 = \dfrac{\frac{20}{(20 + 110)}}{1 - \frac{90}{(90 + 10)}}$

**D** $9{,}82 = \dfrac{\frac{90}{(90 + 20)}}{\frac{10}{(10 + 110)}}$

**E** The probability ratio cannot be calculated from this information

***Answer 2:*** _____

## QUESTION 3

The trainee is now very enthusiastic, claiming more about the patients in the numerical example (question 2):

**1)** Without any further information, it can be stated with a 90 per cent probability that the sample patients with pathological ultrasound findings have salivary gland inflammation.

**2)** Without any further information, it can be stated that for the sample patients with normal ultrasound findings the probability of a false finding is 18% → [20÷(20+90)].

**3)** The positive predictive value is calculated directly from the quotient of the probability ratios → [90%=0.09÷0.01].

*You tell him:*

**A** All statements (1, 2, 3) are incorrect.
**B** The first statement (1) is correct; the other statements (2, 3) are incorrect.
**C** The second and third statements (2, 3) are correct; the first statement (1) is incorrect.
**D** The first and third statements (1, 3) are correct; the second statement (2) is incorrect.
**E** All statements (1, 2, 3) are correct.

***Answer 3:*** _____

## QUESTION 4

A pharmaceutical representative visits you in your practice and introduces you to a new drug that in a large double-blind randomised controlled study on healthy employees of the pharmaceutical company - has achieved a fifty per cent reduction in the risk of dying of a salivary gland carcinoma (mucoepidermoid tumour):

- 4,000 people were treated, of whom 4 (0.1%) died from salivary gland carcinoma.
- Of the 4,000 untreated controls, 8 (0.2%) died.

The pharmaceutical representative, therefore, recommends treating all patients with the new drug. You want to save the life of at least one patient in your practice, but find that you have to treat the following number of patients to achieve this:

**A** 1,000 patients *[= 1÷(0.2%-0.1%)]*
**B** 2,000 patients *[= 8,000÷4]*
**C** 4,000 patients *[= 4 •(1÷0.1%)]*
**D** 8,000 patients *[= 4,000•2]*
**E** cannot calculate the number of patients that you need to treat

***Answer 4:*** _____

## QUESTION 5

You enjoy this opportunity to help humankind and call a colleague to share the good news. Your colleague has already read the publication of the study data (question 4) and points out the following number of cases of pulmonary oedema of unclear origin:

- 7 in the treatment group
- 2 in the control group

*You check and find that one additional pulmonary oedema can be expected when the following number of patients is treated:*

**A** 1,000 treated patients [= 2,000•0.2%]
**B** 571 treated patients [= 4,000÷7]
**C** 800 treated patients [= 1÷(5÷4,000)]
**D** 2,000 treated patients [= 2•(1÷0.1%)]
**E** The number of patients to be treated cannot be calculated from this data.

***Answer 5:*** _____

## QUESTION 6

A patient with chronic toothache recently read in her TV magazine that exposure to formaldehyde could cause chronic toothache. She now wants the health insurance to fund her move. Therefore, she asks whether connection between chronic toothache and formaldehyde in indoor air has been scientifically proven. You do a literature search and find various studies on this topic.
Which study design do you think would be the most appropriate for studying this question?

**A** Prevalence study
**B** Ecological study
**C** Case- control- study
**D** Prospective- randomised controlled study
**E** Case- report

***Answer 6:*** _____

**QUESTION 7**

In detail, the following procedures have been chosen for the studies you have found. Which do you think would be the best way to check whether there is a connection between toothache and formaldehyde in the room?

**A** Measure the formaldehyde content in the living quarters of 100 patients from a specialised ambulance who have a toothache and 100 patients from general dental practices without a toothache, but who correspond to the toothache patients in age, gender and income.

- **Compare the mean formaldehyde content in both groups.**

**B** Interview 500 patients at an environmental medicine ambulance, and ask whether they have a toothache and whether their homes are exposed to formaldehyde.

- **Compare the frequencies of the two answers (formaldehyde-stressed living rooms) from patients with a toothache vs patients without a toothache.**

**C** Interview tenants from a housing company using a toothache questionnaire. Simultaneously perform a skin test for formaldehyde allergy.

- **Compare the incidence of toothache in tenants with formaldehyde allergy vs remaining tenants.**

**D** Perform a two-time measurement of the formaldehyde concentration in the blood of toothache patients from a pain clinic at a one-year intervals.

- **Compare the values at the beginning with those at the end of the observation.**

**E** Survey, using a toothache questionnaire, persons who have newly moved into presumably formaldehyde-contaminated living quarters of an urban housing company and long-term tenants of such apartments.

- **Compare the frequency of toothache between new and long-term tenants.**

***Answer 7:*** _____

## QUESTION 8

A large study has investigated whether a new antibacterial mouthwash reduces mortality from bacterial endocarditis.
The study involved 1,000 high-risk patients. At random, 500 patients were placed in the treatment group and 500 in the control group. The treatment group received the antibacterial mouthwash for over a year, while the control group received a similar-looking mouthwash (as a placebo). The patients in the treatment group were examined monthly for the occurrence of side effects while the patients in the control group were examined only twice a year.
In the treatment group, the number of patients who died of bacterial endocarditis was 10 less than in the control group ($p < 0.01$). Consider whether the quality of the study will convince you.

The following statement is most likely to apply:

**A** The study was not conducted prospectively.
**B** The study was not randomised.
**C** The study was not performed double-blind.
**D** The study did not investigate the issue endpoint (which was interesting for the study) but rather a surrogate endpoint.
**E** In this prospective randomised-controlled double-blind study, the endpoint of interest for the research question was investigated.

***Answer 8:*** _____

## QUESTION 9

1) From the available information (question 8), it is possible to calculate how many patients, as in the study, have to be treated with the antibacterial mouthwash in order to prevent an additional endocarditis death.

2) In order to calculate the number of patients that need to be treated to prevent an additional death, one must know the relative reduction in the risk of death of the treatment group over the control group.

3) The available data (question 8) indicate the relative reduction in the risk of death of the treatment group compared to the control group (relative risk reduction).

**A** All statements (1, 2, 3) are incorrect.
**B** The first statement (1) is correct; the other statements (2, 3) are incorrect.
**C** The second and third statements (2, 3) are correct; the first statement (1) is incorrect.
**D** The first and third statements (1, 3) are correct; the second statement (2) is incorrect.
**E** All statements (1, 2, 3) are correct.

***Answer 9:***_____

## QUESTION 10

You are a specialist in oral and maxillofacial surgery. Your 63-year-old female patient has a 70% stenosis of the left facial artery as an incidental finding. You are wondering whether this is an indication for referral for endarterectomy. You find a study that shows no benefit from surgery for conservative treatment in asymptomatic patients with 70% stenosis (comparable to your patient) at 5 years follow-up. In a subgroup analysis (13 subgroups in total), after taking into account the risk factor status at baseline, only those women who survived the first year without insult or TIA showed over the following 4 years a statistically significant benefit ($p < 0.03$) from endarterectomy.

*Which statement is correct?*

**A** The significant result demonstrates a benefit to women and is sufficient alone to justify the indication for surgery.
**B** In the subgroup, analysis has been corrected for other risk factors, which usually leads to misleading conclusions.
**C** Subgroup analyses maximise the yield of reliable results from randomised controlled trials.
**D** As the number of subsequently formed subgroups increases, there is an increased danger that a subset will erroneously find a benefit that does not exist.
**E** The subgroup analysis, in this case, shows that asymptomatic facial artery stenosis is more dangerous for women than for men.

***Answer 10:*****_____**

## QUESTION 11

In an education course on the effects of medications on oral cancer, the following randomised controlled trials are presented. Various medications were used in them. All studies included a placebo group with middle-aged (55-year-old) patients with normal oral hygiene and tobacco consume. The medications were tested against a placebo over a period of 5 years.

1) In a Bolivian study, therapy II reduced the risk of fatality by 25%.
2) In an Argentinian study, therapy I lowered the risk of fatal outcomes by 30%.
3) In a Chilean study, 3% of the patients in the treatment III group and 4% in the control group died from oral cancer.

*Which statement is correct?*

A Therapy 1 is preferred because this therapy reduces the risk of death the most.
B Therapy 3 is preferred because the majority of her patients benefit from the treatment.
C For therapies 2 and 3, the relative reduction in the risk of death is the same.
D For therapies 1 and 3, the relative reduction in the risk of a fatal outcome is the same.
E None of the therapies indicates the risk for untreated patients (control event rate).

***Answer 11:*****_____**

## QUESTION 12

In a maxillofacial clinic, the prevalence of squamous cell carcinomas is 30%. One thousand consecutive patients will be included in a study for a new, non-invasive, low-stress diagnostic test for the detection of squamous cell carcinoma. The test detects 630 patients as being truly negative, properly tumour free. The number of false-negative and false-positive patients is identical.

| | **Gold standard positive** | **Gold standard negative** | |
|---|---|---|---|
| **Test positive** | | | |
| **Test negative** | | | |
| | | | |

*(4-field table for your own calculation of the result)*

Which 4-field table corresponds to this information?

***Four-field-Table A***

| | *Gold standard positive* | *Gold standard negative* | |
|---|---|---|---|
| *Test positive* | 230 | 70 | 300 |
| *Test negative* | 70 | 630 | 700 |
| | 300 | 700 | 1000 patients |

***Four-field-Table B***

| | *Gold standard positive* | *Gold standard negative* | |
|---|---|---|---|
| *Test positive* | 300 | 0 | 300 |
| *Test negative* | 0 | 700 | 700 |
| | 300 | 700 | 1000 patients |

***Four-field-Table C***

| | *Gold standard positive* | *Gold standard negative* | |
|---|---|---|---|
| *Test positive* | 270 | 100 | 370 |
| *Test negative* | 30 | 600 | 630 |
| | 300 | 700 | 1000 patients |

***Four-field-Table D***

| | *Gold standard positive* | *Gold standard negative* | |
|---|---|---|---|
| *Test positive* | 670 | 30 | 700 |
| *Test negative* | 30 | 270 | 300 |
| | 700 | 300 | 1000 patients |

***Four-field-Table E***

| | *Gold standard positive* | *Gold standard negative* | |
|---|---|---|---|
| *Test positive* | 300 | 70 | 370 |
| *Test negative* | 70 | 560 | 630 |
| | 370 | 630 | 1000 patients |

***Answer 12:_____***

**QUESTION 13**

Some time ago, you referred a 40-year-old female patient to a maxillofacial surgeon because of a palpable nodule in the vestibule. The nodule was classified as benign after the puncture. The patient brings her findings and wants to know whether the cyst means she’s at particular risk of developing cancer. You find several studies. Which study is most suitable for assessing the prognostic significance of benign cysts in patients from a normal population?

**A** A study from the Maxillofacial Surgery Clinic of a Paris university hospital: From 1996−1998. All patients with carcinoma who were found to have palpable cysts were interviewed. For comparison, patients without carcinoma were examined for palpable cysts. Patients with carcinoma had 20% fewer cysts than patients without carcinoma.

**B** A study from a university hospital in Boston: Radiographs (X-rays) from 1,500 patients spanning the last 10 years and showing carcinoma retrospectively examined for cysts. Thirty per cent of the patients also had larger cysts.

**C** A study from the ambulance of the only referral centre in Eastern Scotland for patients with problems in the vestibule: All patients with palpable cysts were examined 10 years later or followed through the cancer registry and death records. Compared with the normal population, the frequency for carcinomas was twice as high.

**D** A study from the pathology tissues at a special clinic in the Ruhr area: All tissue histology from the last 10 years was recorded. Eleven per cent of patients with one benign cyst were also diagnosed histologically with carcinoma within the detection period.

**E** A multicentre study in several district hospitals: Experienced surgeons were asked how often carcinoma patients were diagnosed with benign cysts. The mean of the information was 37%.

***Answer 13:*_____**

**QUESTION 14**

In German-speaking countries, a free-floating thrombus is particularly feared in deep vein thrombosis because of the presumed higher embolic risk. You would like to know whether patients with a free-floating thrombus are at a higher risk for pulmonary embolism than patients with a wall-mounted thrombus.

**1)** This is a question about a forecast.
**2)** This is a question about side effects.
**3)** This question is best examined in a case- control study.
**4)** This question is best studied in a cohort study.
**5)** This question is best studied in a randomised controlled trial.

*Which statement is correct?*

**A** 1 and 3 are correct
**B** 1 and 4 are correct
**C** 1 and 5 are correct
**D** 2 and 4 are correct
**E** 2 and 5 are correct

***Answer 14:***_____

**QUESTION 15:**

A meta-analysis is a type of study that has gained increasing importance lately. Which statement is correct?

**A** Through the technique of meta-analysis, large studies with many patients (mega-trials) have become less significant.

**B** Systematic studies have shown that studies conducted in the respective national language are worse than the studies in English magazines. For this reason, it is sufficient for a meta-analysis to consider the view of English-language literature.

**C** Studies with larger numbers of patients often find a greater therapeutic effect than studies with a smaller number of patients.

**D** In a meta-analysis of randomised trials with a high number of patients, the effectiveness of measures can be estimated more accurately than in smaller individual studies.

**E** In a meta-analysis, there may be differences in the question or the patient populations of the individual studies may be balanced by using statistics.

***Answer 15:***_____

- END OF PART A -

# *- BEGINNING PART B -*

## QUESTION 1

You are a dentist on the maxillofacial surgical ward of a rural hospital (primary care). A 46-year-old female patient underwent this week to remove a small oral tumour (pT1 pN0 M0). The patient is distressed due to the recent diagnosis and wants to know how long she will have to live without further treatment. You are not sure if and to what extent the life expectancy of the patient is limited after successful surgery without follow-up treatment. You want to answer the question using suitable studies.

*Which type of study is best for answering this question?*

**A** Case- control-study
**B** Cross- sectional study
**C** Cohort- study
**D** Case- report
**E** Prevalence study

***Answer 1:***_____

## QUESTION 2

In your literature search, you come across the following studies. Which one is best for answering the question of the patient (see Question 1)?

A In a large multi-centre study, 3,600 women were randomised into two groups after surgical removal of oral floor cancer. One group received irradiation after; the other received adjuvant chemotherapy. The survival rates of both groups were given.

B In a southern German town, all women over 40 were asked whether they had had an oral mucosal surgery. For the group of women with oral cancer surgery in the anamnesis, the mortality rate for the different tumour stages was calculated from the age distribution.

C The now- retired head of your department had for years collected cases on women with T1 carcinomas on whom he had operated. His experiences were published in a special volume on his 65th. Not a single patient had died prematurely.

D In a German city- state, all maxillofacial surgical clinics had joined together to form a study group. All women who had undergone surgical removal of oral floor cancer were registered with their tumour stage at the time of surgery. At annual intervals, it was checked whether the women are still alive. Survival rate was grouped by tumour stages.

E The pathological institute of a renowned German university clinic had registered all cases in which oral cancer was operated on in the prehistory. The survival rate is calculated from the difference between the surgery date and date of death and grouped according to tumour stage.

***Answer 2:***_____

**QUESTION 3**

A patient read an ad for immunotherapy in a US women's magazine. She wants to know whether immunotherapy is a proven medication.

**1)** For detecting drug effects, a double-blind, randomised controlled trial is a suitable study type.
**2)** Randomisation should lead to an approximately equal distribution of patient characteristics between treatment and control groups.
**3)** By randomising, unknown factors that may be important for healing are equally likely to be effective in both groups.

**A** All statements (1, 2, 3) are incorrect.
**B** The first statement (1) is correct; the other statements (2, 3) are incorrect.
**C** The second and third statements (2, 3) are correct; the first statement (1) is incorrect.
**D** The first and third statements (1, 3) are correct; the second statement (2) is incorrect.
**E** All statements (1, 2, 3) are correct.

***Answer 3:***_____

**QUESTION 4**

In a study on the efficacy of immunotherapy for squamous cell carcinoma (PEC), a total of 10,000 women from 37 US clinics were included immediately after the surgical removal of a PEC. Each clinic has a list of the numbers generated by a random number generator. When a patient was enrolled, the next unused number was assigned to the patient's list and then crossed out. In the case of an even number, the patient was treated with 2x30 mg of immunotherapy daily for three years. In the case of an odd number, the corresponding patient was observed over the same period but not treated. At the end of the three-year observation period, the number of patients in the treated and untreated groups who had died was noted. In the treated group, 800 out of 5,000 patients had died within three years while 1,000 out of 5,000 had died in the untreated group.

*The study had the following characteristics*
**1)** Randomised
**2)** Hidden randomised (concealed allocation)
**3)** Double blind
**4)** Controlled
**5)** Multi- centre

*What features does the study have?*
**A** 1, 2 and 5 are correct
**B** 1, 3 and 5 are correct
**C** 1, 4 and 5 are correct
**D** 2 and 3 are correct
**E** 4 and 5 are correct

***Answer 4:***_____

## QUESTION 5

Which statement(s) about the influence of treatment on mortality risk is correct in this study?

**1)** In the treated group, the risk of death decreased by 20% of the risk of the untreated group ***[relative reduction of risk 20% = (1,000-800)÷1,000].***

**2)** In the treated group, the risk of death decreased by 80% of the risk of the untreated group ***[relative reduction of risk 80% = (800÷1,000)].***

**3)** In the treated group, the mortality risk was 2 percentage points below that of the untreated group ***[absolute reduction of risk 2%= (1,000-800)÷10,000].***

**4)** In the treated group, the mortality risk was 4 percentage points below that of the untreated group ***[absolute reduction of risk 4% = (1,000-800)÷5,000].***

**5)** In the treated group, the mortality risk was 10 percentage points below that of the untreated group ***[absolute reduction of risk 10% =1,000÷10,000].***

**A** 1 and 3 are correct
**B** 1 and 4 are correct
**C** 1 and 5 are correct
**D** 2 and 4 are correct
**E** 2 and 5 are correct

***Answer 5:***_____

## QUESTION 6

Assume in this study (question 5), that the relative risk reduction was 50% and the absolute risk reduction was 2%.
How many patients would need to be treated with immunotherapy to prevent an additional death?

**A** *48=50-2*
**B** *50=1÷2%*
**C** *52=50+2*
**D** *100=5,000•2%*
**E** For this calculation, further information is necessary.

***Answer 6:***_____

## QUESTION 7

Whether prolonged medication with an antiallergic drug could reduce the risk of anaphylactic reactions was investigated in a large multicentre, double-blind study. The treatment and placebo groups were exactly the same sizes.
In the placebo group, 25% of patients experienced an anaphylactic reaction. The absolute risk reduction was 2.4% (95% confidence interval = 0.5%–20%).
Please check the following findings

1) By taking the antiallergic treatment, the risk of suffering an anaphylactic reaction was reduced by about 10%.
2) Taking the antiallergic drug treatment in about 2.4% of patients prevented an anaphylactic reaction.
3) The difference between the placebo and antiallergic (treatment) group was statistically significant.

*Which statement(s) apply?*

**A** No statement is correct
**B** 1 is correct
**C** 2 is correct
**D** 2 and 3 are correct
**E** All statements are correct

***Answer 7:_____***

## QUESTION 8

A new test for the detection of dysplasia in oral mucosal lesions has been introduced in the hospital emergency department. The brush biopsy has only two possible outcomes: positive (= dysplasia) or negative (= no dysplasia). The sensitivity is 99%, the specificity is 60%.

1) A diagnostic test with very high sensitivity is well suited for the exclusion of the examined disease (here: dysplasia).

2) High sensitivity means that the diagnostic test will most likely find the disease you are looking for when it exists.

3) In a diagnostic test with very high sensitivity, the probability is very low, that, despite a negative test result (here: no dysplasia), the sought disease (here: dysplasia) nevertheless exists.

**A** All statements (1, 2, 3) are incorrect.
**B** The first statement (1) is correct; the other statements (2, 3) are incorrect.
**C** The second and third statements (2, 3) are correct; the first statement (1) is incorrect.
**D** The first and third statements (1, 3) are correct; the second statement (2) is incorrect.
**E** All statements (1, 2, 3) are correct.

***Answer 8:_____***

**QUESTION 9**

The properties of a brush biopsy were investigated in a study. At a prevalence of 30% for dysplasia, 1,000 consecutive patients were included in the study. The test detects 630 patients as being truly negative, which means properly dysplasia free. The number of false negative and false positive patients is identical.

| | **Gold standard positive** | **Gold standard negative** | |
|---|---|---|---|
| **Test positive** | | | |
| **Test negative** | | | |
| | | | |

(4-field table for your own calculation of the result)

*Which 4-field table corresponds to this information?*

**Four-field-Table A**

| | *Gold standard positive* | *Gold standard negative* | |
|---|---|---|---|
| Test positive | 230 | 70 | 300 |
| Test negative | 70 | 630 | 700 |
| | 300 | 700 | 1000 patients |

**Four-field-Table B**

| | *Gold standard positive* | *Gold standard negative* | |
|---|---|---|---|
| *Test positive* | 300 | 0 | 300 |
| *Test negative* | 0 | 700 | 700 |
| | 300 | 700 | 1000 patients |

**Four-field-Table C**

| | *Gold standard positive* | *Gold standard negative* | |
|---|---|---|---|
| *Test positive* | 270 | 100 | 370 |
| *Test negative* | 30 | 600 | 630 |
| | 300 | 700 | 1000 patients |

**Four-field-Table D**

| | *Gold standard positive* | *Gold standard negative* | |
|---|---|---|---|
| *Test positive* | 670 | 30 | 700 |
| *Test negative* | 30 | 270 | 300 |
| | 700 | 300 | 1000 patients |

**Four-field-Table E**

| | *Gold standard positive* | *Gold standard negative* | |
|---|---|---|---|
| *Test positive* | 300 | 70 | 370 |
| *Test negative* | 70 | 560 | 630 |
| | 370 | 630 | 1000 patients |

***Answer 9:_____***

## QUESTION 10

Another study examined the suitability of the test for diagnosis or excluding lichen planus. Examination of 800 patients with and without lichen planus revealed the following results:

| | | Actual diagnosis Lichen planus | |
|---|---|---|---|
| | | Yes | No |
| **Chairside-test-results** | Lichen planus | 90 | 210 |
| | No lichen planus | 110 | 190 |

In this test, the probability that a patient you know nothing about will actually have lichen planus if the test is positive ("Lichen") (positive predictive value) is:

**A** *50%=(90+110)÷(210+190)*
**B** *30 =90÷(90+210)*
**C** *45%=90÷(90+110)*
**D** *100% =110+190) ÷(90+210)*
**E** *27.5%= 110÷(210+190)*

***Answer 10:***_____

## QUESTION 11

The test is used for the first time in the emergency room with an older man. You are not at all sure if he has lichen planus or not. Therefore, based on your clinical impression, you estimate the likelihood of lichenification to be 50%. The resulting brush biopsy test is positive. You know from the above study that the probability ratio for a positive finding is 7.7 and for a negative finding 0.3. Therefore, you now estimate the probability that lichen planus is present at:

**A** About 90%
**B** About 75%
**C** About 60%
**D** About 45%
**E** A statement is not possible before performing an excisional biopsy (An unlabeled Fagan nomogram is included)

***Answer 11:***_____

## QUESTION 12

The lab supervisor suggests that the dysplasia determination can also be done with more accurate histology in his lab because, in the laboratory, the number of dysplastic cells can be precisely determined and given as a numerical value. As a result, the significance should be greater than in the case of the chairside test, which provides only positive or negative results. The laboratory supervisor emphasises his argument using a study in which the number of dysplastic cells was divided into five levels.
A total of 2,579 patients with and without dysplasia had the following results:

| **Result level** | **Dysplastic cells** | **Patients with lichen planus** | **Patients without lichen planus** |
|---|---|---|---|
| *High positive* | > 55/dl | 474 (59%) | 20 (1.1%) |
| *Moderate positive* | 40 – 55/dl | 175 (22%) | 79 (4.5%) |
| *Neutral* | 25 – 39/dl | 82 (10%) | 171 (10%) |
| *Moderate negative* | 10 – 24/dl | 30 (3.7%) | 168 (9.5%) |
| *Strong negative* | < 10/dl | 48 (5.9%) | 1332 (75%) |
| | Total: | 809 (100%) | 1770 (100%) |

The likelihood ratio (= likelihood ratio) for a moderately positive result (40-55 / dl) in this study is:

A $4{,}8 = \dfrac{\frac{175}{809}}{\frac{79}{1770}}$

B $1{,}0 = \dfrac{\frac{175}{79}}{\frac{1770}{809}}$

C $0{,}02 = \dfrac{\frac{20}{474}}{\frac{175}{79}}$

D $0{,}01 = \dfrac{\frac{30+48}{168+1332}}{\frac{474+175}{20+79}}$

**E** The probability ratio cannot be calculated with this information

***Answer 12:*****_____**

## QUESTION 13

Meta-analysis is an important method for summarising different studies. Which features reduce the credibility of a meta-analysis in most cases?

1) A separate evaluation of studies with significant and non-significant results
2) A separate evaluation of randomised and non-randomised trials
3) Restriction to randomised trials
4) Restriction to English-language publications
5) Restricting publications to only one of several conceivable therapeutic approaches

*The credibility of a meta-analysis is usually reduced by the following properties:*

**A** 2
**B** 1 and 4
**C** 2 and 4
**D** 1, 2, 4 and 5
**E** All

***Answer 13:***_____

## QUESTION 14

The use of mobile phones is repeatedly discussed as a health risk. In the department of maxillofacial surgery of your hospital several cases of the rare (worldwide annual incidence below 1/100,000) malignant ear cancer (abbreviated: EBOK) in mobile users were diagnosed in a short time. The chief physician will ask you whether it has been scientifically proved that mobile phone use causes EBOK. You immediately start a literature search to answer this question. Which study design do you think would be best for studying this question?

**A** Prevalence study
**B** Ecological study
**C** Case- control- study
**D** Prospective randomised controlled study
**E** Case- report

***Answer 14:***_____

## QUESTION 15

You will find the following studies in your literature search. Which one is best for answering the question (question 14):

**A** In France, cases of EBOK were sought through newspaper advertisements and contacting specialists. Each EBOK patient was assigned four patients from the practice of the attending physician. The assigned patients matched the sex, age and income of the EBOK patients but did not have EBOK. The proportion of mobile users (current and past) in EBOK patients and non-EBOK patients was compared.

**B** In a Burmese provincial capital, the administration (240 people) was equipped with mobile phones. Mobile phones are otherwise strictly prohibited in Myanmar (=Burma). After 5 years, all administrative employees were examined for EBOK. The incidence of EBOK in these individuals was compared to the incidence of EBOK in the general resident population of the province.

**C** A cancer registry in eastern Germany records all cancer deaths. Changes in the number of EBOK cases between 1980 and 1989 and were compared with those between 1990 and 1999 was compared.

**D** In Italy, the incidence of EBOK cases in northern and southern Italy was recorded in 1996. The difference in incidence between the two regions was compared to the difference in per-capita mobile call minutes between the two regions.

**E** In a survey of the majority of the chief physicians of German Max Planck Centres, it was ascertained to what proportion, in the opinion of the respondents, EBOK was due to the use of mobile phones among their patients.

***Answer 15:*** ____

- END OF PART B -
